# Bispecific antibodies targeting B‐cell maturation antigen in multiple myeloma: Current clinical applications, challenges and future directions

**DOI:** 10.1002/cncr.70641

**Published:** 2026-11-04

**Authors:** Enrica Manzato, Gloria Cocchetto, Simona Barbato, Michele Puppi, Simone Masci, Elena Zamagni

**Affiliations:** ^1^ IRCCS Azienda Ospedaliero‐Universitaria di Bologna, Istituto di Ematologia “Seràgnoli” Bologna Italy; ^2^ Department of Medical and Surgical Science University of Bologna Bologna Italy

**Keywords:** B cell maturation antigen (BCMA), bispecific antibodies, multiple myeloma, T‐cell–redirecting therapies

## Abstract

Anti‐B cell maturation antigen (BCMA) therapies, including antibody‐drug conjugates, chimeric antigen receptor T cells, and bispecific antibodies (bsAbs), have recently revolutionized the treatment landscape of multiple myeloma. This review outlines the biologic rationale for targeting BCMA and sheds light on the mechanism of action of bsAbs and the main reasons behind loss of response and treatment resistance. Moreover, available safety and efficacy information on approved agents (i.e., teclistamab, elranatamab, and linvoseltamab) are described, focusing on the most relevant treatment‐related toxicities, including cytokine release syndrome, immune effector cell‐associated neurotoxicity syndrome, cytopenias, and infections, and established approaches for their monitoring and management. Furthermore, available data on active trials evaluating the use of bsAbs in combination with other agents and/or in earlier lines of therapy are discussed, as well as emerging data on novel anti‐BCMA bsAbs in clinical development, such as etentamig. Finally, the use of bsAbs beyond multiple myeloma has been explored by reviewing clinical trials and real‐world experiences in other monoclonal gammopathies (e.g., smoldering multiple myeloma and light‐chain amyloidosis) or other conditions, such as autoimmune diseases. Overall, the objective of this review was to provide a comprehensive overview on the role of BCMA‐targeting bsAbs while discussing future developments in this field, including their use across different settings and combinations strategies, as well as the ongoing efforts to improve safety and tolerability through the implementation of specific guidelines for toxicity management.

## Abstract 2

Anti‐B cell maturation antigen therapies, including antibody‐drug conjugates, chimeric antigen receptor T cells, and bispecific antibodies, have recently revolutionized the treatment landscape of multiple myeloma. This review outlines the mechanism of action of anti‐B cell maturation antigen bispecific antibodies in multiple myeloma and the main reasons behind loss of response and treatment resistance, with the objective of providing a comprehensive overview, while discussing future developments and their use across different settings.

## INTRODUCTION

Multiple myeloma (MM) is the second most common hematologic malignancy, accounting for 10%–15% of all hematologic tumors.^1^ Its prognosis has markedly improved thanks to the introduction, since the early 2000s, of novel drugs (anti‐CD38 monoclonal antibodies, proteasome inhibitors, immunomodulatory agents)^1^, ^2^; however, the disease still typically goes through periods of remission followed by relapses, requiring a switch in therapy.^1^

Until recently, patients exposed to all three classes of novel agents (triple‐class exposed [TCE]) had very limited treatment options.^3^ An answer to this relevant, unmet need was provided by developing therapies targeting the B‐cell maturation antigen (BCMA), including T‐cell–redirecting therapies (TCRts), that is, chimeric antigen receptor (CAR) T cells and bispecific antibodies (bsAbs), and antibody–drug conjugates, such as belantamab mafodotin. Currently, two anti‐BCMA CAR T‐cell therapies (idecabtagene vicleucel and ciltacabtagene autoleucel), three anti‐BCMA bsAbs (teclistamab, elranatamab, and linvoseltamab), and two belantamab‐based combination therapies are recommended in relapsed/refractory MM.^4^

This review focuses on anti‐BCMA bsAbs in MM, either approved or under study, describing their mechanisms of action, main information on their efficacy and safety, and the most relevant future developments, including earlier phases of the disease, other monoclonal gammopathies, and conditions.

## FOCUS ON BIOLOGY: BCMA IN NORMAL AND MALIGNANT PLASMA CELLS, bsAbs STRUCTURE AND MECHANISM OF ACTION, AND REASONS FOR RESISTANCE

### BCMA in healthy plasma cells and MM cells

BCMA was first identified in the 1990s^5^ and was then detected on mature B cells, including plasma cells (PCs)^6^, ^7^, ^8^; its expression has been reported on plasmacytoid dendritic cells,^9^ keratinocytes,^10^ adipocytes,^11^ and glioma cells,^12^ although mainly identified as B‐cell lineage–specific.^6^, ^7^, ^8^ It has since been characterized as a membrane receptor for two ligands belonging to the tumor necrosis factor (TNF) family, B‐cell–activating factor (BAFF) and the A proliferation‐inducing ligand (APRIL), playing a role in the survival of mature B cells and bone marrow PCs.^13^, ^14^, ^15^ In addition, a soluble form (sBCMA) shed by PCs through cleavage of the extracellular domain by γ‐secretase^8^, ^16^ also binds APRIL^16^ and possibly BAFF.^16^, ^17^

BCMA membrane expression is maintained in MM cells^6^, ^18^, ^19^, ^20^, ^21^ despite variations across samples^22^ and contributes to a series of protumor effects, including: cell survival and proliferation^18^; resistance to apoptosis induced by dexamethasone and lenalidomide^19^, ^20^; adhesion to BAFF‐secreting bone marrow stromal cells, creating a positive feedback loop^20^, ^21^; and upregulation of angiogenesis‐related genes, immune suppression, and osteoclast activation.^21^ Its expression has also been reported in other hematologic malignancies involving the B‐cell line and in acute myeloid leukemia.^23^

The ubiquitous and highly specific expression of BCMA on PCs, together with its important role in MM cell survival and proliferation, have made it an attractive therapeutic target. Similarly, sBCMA likely contributes to immune deficiency and reduced antibody production by preventing BAFF binding to normal B cells and PCs.^17^ Furthermore, higher concentrations are found in patients versus healthy controls, as well as active disease versus remissions, correlating with shorter overall survival, thereby representing a potential prognostic and predictive factor.^24^, ^25^

### BsAb structure and mechanism of action

BsAbs are designed with two different antigen‐binding sites, one for a tumor‐specific antigen and one for T cells (typically CD3), to bring them closer and form an immunologic synapse activating T cells.^26^, ^27^ This interaction is independent from the major histocompatibility complex and thus is not antigen‐specific,^26^, ^27^ although costimulatory signaling from major histocompatibility complex class I molecules also plays a role.^28^ Once the synapse has been established, a series of downstream effects occur, most notably a cytotoxic activity with perforin and granzyme secretion against the tumor cell but also with T‐cell differentiation and proliferation and cytokine secretion.^26^, ^27^, ^29^, ^30^, ^31^ CD8‐positive T cells are the main cells undergoing expansion^28^ and involved in direct cytotoxicity, whereas CD4‐positive helper T cells produce cytokines to enhance the activity of other immune cells, with the downside of contributing to cytokine‐release syndrome (CRS).^27^

It is important to note that tumor antigens that are larger and farther from cell membrane reduce the immunologic synapse efficiency and T‐cell activation, whereas higher bsAb affinity for the target usually translates into higher efficacy. Also, different CD3 affinities affect bsAb biodistribution and CRS incidence (with lower affinities generally reducing CRS but sometimes compromising efficacy), and bsAb biochemical structure has an important effect on pharmacokinetics and side effects.^27^ Indeed, bsAbs are typically divided into two categories based on the presence or lack of a fragment crystallizable (Fc) region: those with an Fc region typically resemble immunoglobulin G (IgG) molecules, but they can also contain additional binding sites.^32^ The Fc region preserves antibody‐dependent, cell‐mediated phagocytosis and cellular cytotoxicity, complement fixation, and recycling through the neonatal Fc receptor (FcRn).^32^, ^33^ However, although FcRn recycling extends the bsAb’s half‐life, other functions have been associated to unwanted toxicities and reduced cytotoxicity, so Fc domains are usually engineered to maintain FcRn binding but limit others.^26^

### Loss of response and resistance to anti‐BCMA bsAbs

BCMA bsAbs are not immune from primary resistance or disease relapse, with different mechanisms thought to come into play in primary resistance (e.g., sBCMA, high tumor burden or extramedullary disease [EMD] with an inadequate effector‐to‐target ratio) or secondary resistance (e.g., antigen loss or mutation), albeit with some overlap (e.g., immune system dysfunction is involved in both kinds of resistance). Some clinical factors that predict a poor response are: previous anti‐BCMA therapy, International Staging System stage III disease, high tumor burden, and EMD.^34^, ^35^, ^36^ It has also been observed that sBCMA levels are correlated with worse outcomes of teclistamab therapy and are associated with features of high‐risk disease.^35^, ^37^, ^38^

The most straightforward mechanism of secondary resistance consists in antigen loss, achieved, for instance, through a biallelic gene deletion.^39^, ^40^ In addition, mutations in the extracellular domain arising during therapy preserve BCMA expression but decrease binding affinity, resulting in resistance to some bsAbs, but not others.^41^, ^42^ In addition, MM cells can acquire resistance in a BCMA‐independent manner by differentially regulating other signaling pathways.^28^, ^43^

With regard to primary resistance, it is believed that sBCMA plays a role because it can interfere with anti‐BCMA antibody binding, likely by acting as a decoy receptor.^30^, ^37^, ^44^ Conversely, treatment with an inhibitor of γ‐secretase results in lower sBCMA and improved efficacy.^30^, ^37^, ^45^ In addition, increasing bsAb concentration limits this effect, underlining the importance of an adequate effector‐to‐target ratio.^37^ This issue is particularly evident in patients with EMD or high tumor burden: in preclinical models, high tumor burden was associated with worse response,^46^ whereas retrospective data correlated debulking chemotherapy with better outcomes in patients with EMD or high tumor burden, likely attributable to a better effector‐to‐target ratio and potential immunomodulatory effect of cyclophosphamide (the most common chemotherapy regimen).^47^

Indeed, the immune microenvironment is another main determinant of resistance and has been shown to be involved in determining both primary and secondary resistance. A dysfunctional T‐cell phenotype at the start of therapy has been associated with poor response and shorter progression‐free survival (PFS)^28^, ^38^; in addition, in preclinical models, it was observed that markers of T‐cell dysfunction increased during treatment, possibly leading to a loss of effectiveness,^46^ whereas treatment‐free intervals improved T‐cell expansion, cytotoxic activity, and tumor control.^48^

## CURRENTLY APPROVED ANTI–BCMA BISPECIFIC ANTIBODIES: TECLISTAMAB, ELRANATAMAB, AND LINVOSELTAMAB

Currently approved anti‐BCMA bsAbs for relapsed/refractory MM are teclistamab, elranatamab, and linvoseltamab: the European Medicines Agency approved their use after at least three prior therapies in patients who had TCE disease that progressed on last therapy^49^, ^50^, ^51^; and the US Food and Drug Administration approved their use as monotherapy in patients with TCE disease after four prior lines and has recently approved the combination of teclistamab and daratumumab after one line of therapy that includes an immunomodulatory agent and a proteasome inhibitor.^52^, ^53^, ^54^ All are humanized or fully human IgG antibodies, administered either subcutaneously (teclistamab and elranatamab) or intravenously (linvoseltamab), with two step‐up doses and then continuing with weekly full doses. The schedule can then be reduced to once every 2–4 weeks based on treatment duration and depth of response.^49^, ^50^, ^51^ Main data on their efficacy and safety, as recorded in clinical trials and in real‐world practice, are detailed in Tables 1 and 2, respectively.^35^, ^36^, ^55^, ^56^, ^57^, ^58^, ^59^, ^60^, ^61^, ^62^, ^63^, ^64^, ^65^, ^66^, ^67^, ^68^, ^69^, ^70^

**TABLE 1:** Efficacy data for teclistamab, elranatamab, and linvoseltamab in clinical trials and real‐world studies.

Trial: Reference(s)	bsAb	No. of patients	Study phase	mLoT, no. (range)	HR, %^a^	EMD, %	TCE/TCR, %	Penta exp/ref, %	TCRt exp/ref, %^b^	ADC, exp/ref, %^b^	Aged ≥75 years, %	ECOG PS 0–1, %	RI, %	mFU, months	ORR, %	CR, %	Negative MRD eval, no. (%)	mPFS, months	mOS, months
Evidence from clinical trials																			
MajesTEC‐1, NCT03145181 and NCT04557098: Moreau 2022,^35^ Garfall 2024^55^	Tec	165	1/2	5 (2–14)	26.0	17.0	100.0/77.6	70.3/0.3	0.0/0.0	0.0/0.0	14.5	100.0	0.0	30.4	63.0	46.1	48 (56.0)	11.4	22.2
MagnetisMM‐1, NCT03269136: Bahlis 2023^56^	Elra	55	1	5 (2–14)	29.0	31.0	98.2/90.9	78.2/58.2	25.4/NA	14.5/NA	Median, 64 (range, 42–80)	91.0	NA	12.0	63.6	38.2	13 (13.0)	11.8	21.2
MagnetisMM‐3, NCT04649359: Lesokhin 2023,^36^	Elra	123	2	5 (2–22)	25.0	32.0	100.0/96.7	70.7/42.3	0.0/0.0	0.0/0.0	Median, 68 (range, 36–89)	95.0	0.0	28.4	61.0	37.4	NA	17.2	24.6
LINKER‐MM1, NCT03761108: Bumma et al. 2024,^59^ Lee et al. 2026^60^	Linv	117	1/2	5 (2–16)	39.0	16.0	100.0/82.1	76.9/28.2	0.0/0.0	NA/NA	26.5	100.0	0.0	21.3	70.9	52.1	32 (34.0)	18‐month PFS, 60.4%	31.4

Reference(s)	bsAb	No. of patients	Trial‐NE patients, %	mLoT, no. (range)	HR, %^a^	EMD, %	TCE/TCR, %	Penta exp/ref, %	TCRt exp/ref, %^b^	ADC exp/ref, %^b^	Aged ≥75 years, %	ECOG PS 0–1, %	RI, %^c^	mFU, months	ORR, %	CR, %	Negative MRD eval	mPFS, monthss	mOS, months
Evidence from real‐world data from multicentric retrospective studies																			
Uttervall 2026^61^	Tec	113	70.8	6 (2–15)	52.0	15.0	100.0/78.8	88.5/44.2	11.5/NA	28.3/NA	15.0	PS 0, 44.9	11.0	20.7	60.0	26.5	—	9.7	26.3
Tan 2025^62^	Tec	210	71.0	6 (1–20)	50.0	29.0	NA/83.0	NA/44.0	30.9/NA	20.9/NA	23.3	73.0	12.4	5.3	67.0	21	—	6‐month PFS, 53.0%	6‐month OS, 73.0%
Riedhammer 2024^63^	Tec	123	39.0	6 (3–14)	37.0	36.1	NA/92.6	NA/60.2	17.8/NA	19.5/NA	Median, 67 (range, 35–87)	NA	NA	5.5	59.3	22.0	—	8.7	NR
Razzo 2025^64^	Tec	509	89.0	6 (2–18)	54.0	45.0	NA/81.0	NA/38.0	NA/24.5	NA/17.8	23.0	75.0	16.9	10.1	53.0	25.3	—	5.8	12‐month OS, 61.0%
Perrot 2024^65^	Tec	303	46.2	4 (NA)	67.0	12.0	100.0/NA	NA	NA	NA	29.7	91.0	9.9	11.9	68.8	NA		11.3	17
Mohan 2024^66^	Tec	110	NA	6 (3–13)	62.0	44.0	NA/86.0	NA/76.0	22.7/NA	14.8/NA	25.0	NA	NA	3.5	62.0	20.0	—	6‐month PFS, 52.0%	6‐month OS, 80.0%
Dima 2024^67^	Tec	106	83.0	6 (4–17)	59.0	42.0	NA/92.0	NA/64.0	40.5/NA	22.6/NA	Median, 66.5 (range, 35–87)	67.0	13.0	3.8	66.0	29.0	—	5.4	6‐month OS, 70.0%
Dhakal 2026^68^	Tec	101	NA	4 (IQR, 3–4)	38.0	7.0	93.1/65.3	68.3/54.5	7.9/NA	3.9/NA	Median, 65.4 (IQR, 61.6–69.4)	90.0	23.8	13.8	80.2	36.0	—	12‐month PFS, 65.0%	12‐month OS, 80.6%
Portuguese 2026^69^	Elra	130	78.0	6 (4–8)	69.0	22.0	NA/91.0	NA/49.0	46.0/NA	6.3/NA	Median, 71 (range, 39–95)	65.0	6.1	7.5	65.0	36.0	—	4.3	14.6

Abbreviations: ADC, antibody–drug conjugate; BCMA, B‐cell maturation antigen; bsAb, bispecific antibody; CR, complete response; ECOG PS, Eastern Cooperative Oncology Group performance status; Elra, elranatamab; exp/ref, exposed/refractory; eval, evaluable; IQR, interquartile range; Linv, linvoseltamab; mLoT, median lines of therapy; MRD, minimal residual disease; NA, not available; NE, not eligible; NE, nonestimable; ORR, overall response rate; OS, overall survival; Penta, treatment with proteasome inhibitors, immunomodulatory drugs, and anti‐CD38 monoclonal antibodies; PFS, progression‐free survival; Ref, reference; RI, renal impairment; TCE, triple‐class–exposed; TCR, triple‐class–refractory; TCRt, T‐cell–redirecting therapies; Tec, teclistamab.

^a^ High‐risk cytogenetics are defined as the presence of at least one among: del(17p), t(4;14) and t(14;16), except in Perrot et al.^65^ (in which amp1q is also included); Riedammer,^63^, Dima, Dakhal, Portuguese,^67^, ^68^, ^69^ (in which gain/amp1q are also included); and Mohan et al.^66^ (in which gain/amp1q and t[14;20] are also included).

^b^ Prior exposure to anti‐BCMA (TCRt or ADC, respectively).

^c^ Impaired renal function (RI) is defined as creatinine clearance <40 ml per minute by Tan, Perrot, Dakhal^62^ ^,^ ^65^ ^,^ ^68^; and as <30 ml per minute by Moreau et al.,^35^ Lesokhin et al.,^36^ Garfall^55^, Prince^57^, Tomasson^58^, Bumma^59^, Lee^60^, Riedammer^63^, Mohan.^66^

**TABLE 2:** Safety data for teclistamab, elranatamab, and linvoseltamab in clinical trials and real‐world studies.

			All grades/grade ≥3, %						All grades/grades 3‐4/grade 5, %		
Reference(s)	bsAb	No. of patients	CRS	ICANS	Other NT	Neutropenia	Anemia	Thrombocytopenia	Infections	HGG, %	Ig‐supplem, %
Evidence from clinical trials											
MajesTEC‐1, NCT03145181 and NCT04557098: Moreau 2022,^35^ Garfall et al., see comment above in Table 1 ^55^	Tec	165	72.0/1.0	3.0/0.0	NA	72.0/65.0	55.0/38.0	42.0/23.0	79.0/55.0/22.0	15.0	46.0
MagnetisMM‐1, NCT03269136: Bahlis 2023^56^	Elra	55	66.7/0.0	16.4/0.0	NA	75.0/71.0	67.0/51.0	51.0/29.0	75.0/27.0/4.0	NA	33.0
MagnetisMM‐3, NCT04649359: Lesokhin 2023,^36^ Prince,^57^, Tomasson,^58^) Leleu 2023^70^	Elra	123	58.0/0.0	5.0/0.0	NA	50.0/50.0	49.0/37.0	32.0/24.0	71.0/42.0/8.0	93.5	41.0
LINKER‐MM1, NCT03761108: Bumma et al., Lee et al.,^59^	Linv	117	46.0/1.0	8.0/3.0	NA	44.0/43.0	40.0/31.0	NA	75.0/48.0/6.0	NA	NA
Evidence from real‐world: Data from multicentric, retrospective studies											
Uttervall 2026^61^	Tec	113	55.8/1.7	3.5/0.0	PSN, 4.0/0.0; Mot Dys, 1.0/0.0; enceph, 3.0/1.0	35.0/33.0	26.0/17.0	19.0/15.0	71.0/39.0/6.0	NA	81.0
Tan 2025^62^	Tec	210	54.0/0.5	7.0/1.9	NA	NA/40.0	NA	NA/29.0	56.0/22.0/8.0	69.0	62.0
Riedhammer 2024^63^	Tec	123	59.0/2.0	7.0/1.0	NA	55.0/38.0	81.0/32.0	63.0/26.0	55.0/27.0/NA	NA	NA
Razzo 2025^64^	Tec	509	54.0/1.0	11.0/2.0	NA	NA	NA	NA	42.0/22.0/27.0	38.0	38.0
Perrot 2024^65^	Tec	303	NA	NA	NA	NA	NA	NA	NA	NA	61.0
Mohan 2024^66^	Tec	110	56.0/3.5	11.0/4.6	NA	NA	NA	NA	40.0/26.0/1.0	39.0	NA
Dima 2024^67^	Tec	106	64.0/1.0	15.0/3.0	NA	42.0/15.0	50.0/10.0	47.0/8.0	31.0/NA/3.0	NA	42.0
Dhakal 2026^68^	Tec	101	33.0/0.0	9.0/1.0	NA	NA	NA	NA	27.0/2.0/0.0	NA	39.0
Portuguese 2026^69^	Elra	130	40.0/2.3	17.0/2.3	NA	NA	NA	NA	38.0/NA/NA	NA	46.0

Abbreviations: bsAb, bispecific antibody; CRS, cytokine‐release syndrome; Elra, elranatamab; enceph, encephalopathy; HGG, hypogammaglobulinemia; ICANS, immune effector cell–associated neurotoxicity syndrome; Ig‐supplem, immunoglobulin supplementation; Linv, linvoseltamab; Mot Dys, motor dysfunction; NA, not available; NT, neurotoxicity; PSN, peripheral sensory neuropathy; Ref, references; Tec, teclistamab; Thromb, thrombocytopenia.

### Efficacy

The MajesTEC‐1 (ClinicalTrials.gov identifiers NCT03145181 and NCT04557098), MagnetisMM‐1 (ClinicalTrials.gov identifier NCT03269136) and ManetisMM‐3 (ClinicalTrials.gov identifier NCT04649359), and LINKER‐MM1 (ClinicalTrials.gov identifier NCT03761108) trials for teclistamab, elranatamab and linvoseltamab, respectively, have shown encouraging efficacy rates, with 61%–71% overall response rates (ORRs), 37%–52% complete response (CR) rates, and high rates of minimal residual disease (MRD) negativity, when evaluable. The median PFS (mPFS) ranged between 11 months and not reached,^35^, ^36^, ^55^, ^60^ with better results in patients who achieved a CR: for them, the 30‐month PFS rate was 61% with teclistamab,^55^ the 24‐month PFS probability was 79.1% (95% confidence interval [CI], 65.3%–87.9%) with linvoseltamab,^60^ and the probability of maintaining a response at 24 months was 87.9% (95% CI, 73.1%–94.8%) with elranatamab.^57^ The median OS ranged between 21 and 31 months^35^, ^36^, ^55^, ^56^, ^57^, ^58^, ^59^, ^60^ once again with better results for patients in CR (30‐month rate, 74.2% with teclistamab^66^; 24‐month probability, 84.9% [95% CI, 69.9%–92.7%] with linvoseltamab^60^).

However, because these single‐arm phase 1 and 2 trials lacked comparison with standard‐of‐care treatments, post‐hoc analyses were performed. Specifically, teclistamab monotherapy was compared with standard‐of‐care data derived from electronic health records^71^ or patient populations from clinical studies,^72^, ^73^ showing a statistically significant improvement in response rates, PFS, and the time to next treatment.^71^, ^72^, ^73^ For example, when comparing the MajesTEC‐1 population versus the population from one of the LocoMMotion and MoMMent studies (describing the standard of care in patients with TCE MM before bsAbs), the resulting ORRs were 63.0% versus 26.2%, respectively (relative risk, 2.41; 95% CI, 1.80–3.23; *p* < .0001), and PFS was 11.3 versus 4.07 months, respectively (hazards ratio [HR], 0.48; 95% CI, 0.35–0.64; *p* < .0001).^73^ Elranatamab was compared with real‐world data^74^, ^75^ and with the patient populations from the LocoMMotion and MAMMOTH studies,^76^, ^77^ with similar improvements observed in ORR and PFS. Indeed, patients who received elranatamab were 4.85 (95% CI, 2.85–8.23) and 3.24 (95% CI, 1.98–5.32) times more likely to obtain a response compared with patients in the LocoMMotion and MAMMOTH studies, with a mPFS of 17.2 months (95% CI, 9.8 months to nonestimable) in those who received elranatamab and 4.6 months (95% CI, 3.9–5.6 months) in the LocoMMotion study (*p* < .01).^76^, ^77^ Finally, linvoseltamab was compared with real‐world patients who met criteria for the LINKER‐MM1 study and showed statistically significant improvements in ORR, mPFS, and OS: The ORR in the two groups was 69.5% versus 43.4%, respectively (weighted odds ratio, 3.0; 95% CI, 1.9–4.1; *p* < .001), and the mPFS was not reached versus 6.0 months (weighted HR, 0.33; 95% CI, 0.28–0.40; *p* < .001).^78^

Real‐world data, currently available mainly for teclistamab (Table 1), have largely confirmed the response rates seen in trials, albeit with shorter PFS and OS durations, which typically occurs in less selected populations. These data confirm the feasibility of bsAb therapy also in a frailer population, as observed in populations with older age and worse performance status, as well as the presence of renal impairment^61^, ^62^, ^63^, ^64^, ^65^, ^66^, ^67^, ^68^, ^69^—indeed, older age or greater frailty have not been linked to poorer outcomes, suggesting that these therapies can be used, with careful management, even in these populations.^79^, ^80^, ^81^

Of note, a newer and active avenue of research is investigating response to an anti‐BCMA bsAb after a previous anti‐BCMA agent (whether it is another bsAb, a CAR T‐cell agent, or belantamab mafodotin): overall, responses are still frequent but shorter with bsAb therapy after a CAR T‐cell agent or belantamab mafodotin, whereas information on the sequencing of two different anti‐BCMA bsAbs is even scarcer and not particularly encouraging (one retrospective study reported an ORR of only 12.5%).^82^, ^83^, ^84^ Current guidelines highlight the importance of taking into account both patient‐related (e.g., frailty) and disease‐related factors in choosing the first anti‐BCMA therapy, privileging the most effective and feasible one but also keeping in mind the potential need for the others in subsequent lines. According to this view, the general consensus is, where possible, to choose CAR T‐cell therapy before a bsAb, which, in turn, should be used before belantamab mafodotin, and then to perform a washout between different anti‐BCMA therapies.^83^, ^84^

### Toxicities and their management

CRS and immune effector cell‐associated neurotoxicity syndrome (ICANS) are the main bsAb‐related adverse events (AEs) during the first month, whereas cytopenias and infections generally manifest throughout therapy.

#### Cytokine‐release syndrome

CRS is triggered by bsAbs activating T cells, which release cytokines (e.g., interleukin‐2 and interleukin‐6, TNF‐α, interferon‐γ) and involve myeloid cells in a systemic inflammatory reaction. It manifests with fever and systemic symptoms but can evolve into severe manifestations, such as hypoxia and hypotension, if not promptly treated.^85^, ^86^ All approved anti‐BCMA bsAbs, according to the label, require step‐up dosing and premedication of the first doses with acetaminophen, an antihistamine, and steroids.^86^ In addition, inpatient start of therapy was originally required, although outpatient step‐up dosing is becoming progressively more common as experience in managing AEs increases.^87^, ^88^, ^89^ Prophylactic tocilizumab further reduces CRS rates, with no effect on treatment efficacy,^90^, ^91^, ^92^, ^93^ but its use is still under investigation, with a recent expert panel considering its use in the outpatient setting only for patients at higher risk of CRS.^86^, ^89^

When CRS is suspected, treatment should be interrupted and other causes ruled out.^86^ Then, CRS should be graded following the consensus of the American Society for Blood and Marrow Transplantation^86^, ^94^ and treated accordingly. Specifically, for grade 1 CRS, supportive therapy can be sufficient, but early use of tocilizumab^86^, ^95^ or at‐home dexamethasone (in the outpatient setting) can be useful^89^; for higher grade cases, tocilizumab and/or steroid therapy represent the first lines, with other salvage therapies (e.g., anakinra) in resistant and/or severe cases.^86^

#### Immune effector cell–associated neurotoxicity syndrome

ICANS typically occurs during or after CRS and is thought to be determined by diffusion of cytokines and T cells to the central nervous system, with a contribution from myeloid cells, such as monocytes and macrophages. It manifests as a diffuse encephalopathy, which can progress to seizures and intracranial hypertension if not treated promptly.^86^ However, its incidence is low with bsAbs, with any‐grade ICANS recorded in no more than 17% of patients grade 3 or greater in no more than 5% both in trials and in real‐world patients and thus is less of a concern in clinical practice.^35^, ^36^, ^55^, ^56^, ^57^, ^58^, ^59^, ^60^, ^61^, ^62^, ^63^, ^64^, ^65^, ^66^, ^67^, ^68^

Nonetheless, guidelines recommend a baseline evaluation and monitoring using the immune effector cell‐associated encephalopathy score. When ICANS is suspected, treatment should be interrupted, other causes of neurotoxicity ruled out, and an electroencephalogram and brain imaging can be considered.^86^ Grading follows the American Society for Blood and Marrow Transplantation score,^94^ and treatment is decided accordingly: a grade 1 ICANS can be only monitored, unless patients are deemed at high risk of progression, whereas the mainstay of treatment for grade 2 or worse ICANS is steroids, with second‐line therapy (e.g., anakinra) for severe or resistant cases.^86^

#### Cytopenias

Cytopenias during bsAb therapy typically occur early during treatment, although their pathophysiology remains unclear: proposed driving mechanisms include a poor bone marrow reserve in heavily pretreated patients and therapy‐related toxicity, likely caused by cytokine release and the proinflammatory state triggered by bsAbs (explaining why cytopenias are recorded after their administration and improve with dose delays).^96^

Guidelines recommend a complete blood count with differential to be obtained before every administration, at least for the first period. In the presence of cytopenia, other causes should be excluded and supportive care initiated (i.e., granulocyte‐colony–stimulating factor, erythropoietin, thrombopoietin‐receptor agonists; red blood cell or platelet transfusions), together with dose delays for grade 3 or greater cytopenias.^86^ Granulocyte‐colony‐stimulating factor is usually avoided in patients who have CRS or are considered at higher risk of CRS because of some evidence of its association with worse CRS and ICANS in patients treated with TCRts.^86^

#### Infections

The infections reported vary across studies, but it is estimated that well over one half of patients experience an infection during treatment^97^, ^98^, ^99^; a higher risk of all‐grade, but not grade 3 or greater, infections was associated with combination therapy versus monotherapy.^97^ The most common site is the respiratory tract,^98^, ^99^, ^100^, ^101^, ^102^ followed by bacteremia and urinary tract, skin and soft tissues, and gastrointestinal infections.^98^, ^99^, ^102^ The main pathogens are viruses and bacteria,^99^, ^100^, ^101^ with opportunistic pathogens also reported.^99^, ^102^

This increased risk derives from many factors: MM itself and the toxicities of prior treatments; leukopenia, including both neutropenia and lymphopenia; hypogammaglobulinemia; and T‐cell dysfunction.^96^, ^103^ CRS and ICANS and their therapies, particularly steroids, could also play a role, but data are contrasting.^96^, ^98^, ^99^ B‐cell depletion and hypogammaglobulinemia are regarded at least in part as on‐target, off‐tumor effects because BCMA is also expressed on normal B cells and PCs^103^, ^104^; conversely, immunoglobulin supplementation decreases the incidence of infections, with particularly strong evidence for more severe ones.^70^, ^99^, ^104^, ^105^, ^106^, ^107^ In addition, a reduced dosing schedule has a positive effect on infections,^55^, ^57^, ^107^ which is attributed in part improved T‐cell function.^48^

Information on the timing of infections is not always straightforward, also because of the different reporting methods used,^108^ generally speaking; although earlier studies seemed to suggest a progressively increasing, cumulative rate of infections with no clear plateau,^99^, ^100^ more recent experiences both in clinical trials^57^, ^60^, ^109^, ^110^, ^111^, ^112^ and in real‐life^61^, ^101^ describe a higher incidence of all‐grade and grade 3 or greater infections in the first 3–6 months of therapy, which have been attributed in some instances to a delayed start of immunoglobulin supplementation or a switch to a less frequent dosing schedule.^107^

Baseline testing for hepatitis B and C viruses and for human immunodeficiency virus is recommended; the importance of cytomegalovirus baseline testing and subsequent monitoring is less clear.^86^, ^89^, ^113^, ^114^, ^115^ Moreover, patients should receive the influenza, coronavirus disease 2019, pneumococcal, and varicella zoster vaccinations, with some guidelines also recommending the *Haemophilus influenzae* bacteria vaccine.^86^, ^89^, ^113^, ^114^ Antimicrobial prophylaxes should include herpes and *Pneumocistis jiroveci* throughout treatment and until restoration of adequate T‐cell function as well as hepatitis B virus reactivation prophylaxis for those with a previous infection.^86^, ^89^, ^113^, ^114^ Conversely, antibacterial and antifungal prophylaxes are usually recommended only in patients at heightened risk.^86^, ^89^, ^113^, ^114^ Avoidance of neutropenia with supportive therapy and dose delays also contributes to reducing risk.^86^, ^113^, ^114^ Immunoglobulin support is considered of paramount importance,^70^, ^99^, ^104^, ^105^, ^106^ with recent opinion articles and recommendations leaning toward supplementing all patients, regardless of immunoglobulin levels or prior infection history.^89^, ^116^ Finally, reducing the dosing schedule can further limit infections.^49^, ^50^, ^51^, ^89^, ^107^

## ONGOING RESEARCH

### Anti‐BCMA bsAbs in earlier lines of therapy and in combination with other agents

Preclinical data suggest that combining bsAbs with other anti‐MM agents could increase their effectiveness. The best‐studied combination is with daratumumab (an anti‐CD38 antibody representing the current mainstay of MM therapy), which proved to have a direct antitumor effect and enhance teclistamab‐mediated cytotoxicity, likely through its immunomodulatory effects, resulting in a lower frequency of immune‐suppressive cells and a higher rate of effector T cells. These changes were also observed in daratumumab‐refractory cells, suggesting that the immunomodulatory effects of daratumumab exposure outlive its anti‐MM efficacy and supporting the use of teclistamab either in combination with or after anti‐CD38 agents.^117^ Immunomodulatory drugs have also shown synergic effects with bsAbs in inducing cytotoxicity and T‐cell differentiation.^118^, ^119^ Combinations with novel agents have also been tested: treatment with γ‐secretase inhibitors (e.g., nirogacestat) has shown promise in preclinical models,^30^, ^37^, ^45^ and the microenvironment around MM cells can be targeted by inhibitors of immune‐suppressive molecules such as programmed death‐1 (e.g., cemipilimab), CD47 (e.g., mapliparcept), and the immune checkpoint receptor lymphocyte‐activation gene‐3 (e.g., fianlimab). Active trials with anti‐BCMA bsAbs in earlier lines and/or combination therapy are listed in Table 3, and published data summarized in Table 4.^110^, ^111^, ^112^, ^120^, ^121^, ^122^, ^123^, ^124^, ^125^, ^126^, ^127^, ^128^, ^129^, ^130^, ^131^, ^132^, ^133^

**TABLE 3:** Ongoing trials with bispecific antibodies in earlier lines and/or in combination therapies.

Trial	Study design	Estimated no. of patients	Key inclusion and exclusion criteria	Treatment regimens	Primary end point	Status	Estimated completion date
MajesTEC‐4/EMN30, NCT05243797	Phase 3, randomized multicentric	1594	NDMM, TE	Tec, TecR, R as maintenance	PFS and MRD‐negative CR rate	Recruiting	April 2032
MagnetisMM‐7, NCT05317416	Phase 3, randomized multicentric	854	NDMM, TE	Elra, R as maintenance	PFS	Active, not recruiting	October 2029
IFM 2022‐01, NCT06353022	Phase 2, multicentric, multiple arms	130	NDMM, TE	Tec‐R, Tec‐Tal as maintenance	Rate of sustained MRD negativity and conversion from positive to negative MRD	Active, not recruiting	June 2030
IFM 2025‐01/ElLen, NCT06918002	Phase 3, randomized multicentric	824	NDMM, TE	Elra‐R as consolidation, Elra as maintenance, SOC consolidation and maintenance	PFS, MRD negativity rate	Recruiting	May 2036
IFM‐2024‐06/ElMMA, NCT06931704	Phase 2, multicentric, multiple arms	176	NDMM, TE	Elra‐R, DR as maintenance	MRD negativity rate	Not yet recruiting	November 2031
GMMG‐HD10/DSMM‐XX/MajestTEC‐5, NCT05695508	Phase 2, multicentric, multiple arms	160	NDMM, TE	Tec and Tal with DRd or DVRd induction and with D maintenance	AEs	Recruiting	September 2029
LINKER‐MM4, NCT05828511	Phase 1/2, multicentric, single arm	149	NDMM, TE and TI	Linvo	DLTs, AEs, VGPR and MRD negativity rate	Recruiting	November 2035
ImmunoPLANT, NCT06376526	Phase 2, monocentric, single arm	28	NDMM, TE and TI, MRD positive	Linvo fixed duration	MRD negativity rate	Recruiting	August 2027
MajesTEC‐7, NCT05552222	Phase 3, randomized multicentric	1590	NDMM, TI	TecDR, TalDR, DRd	PFS and 12‐month MRD negativity and CR rates	Recruiting	October 2033
MagnetisMM‐6, NCT05623020	Phase 3, randomized multicentric	1116	NDMM, TI	Elra‐DR, DaraVRd	DLTs, PFS, MRD negativity rate	Recruiting	October 2033
IFM 2021‐01/TecLille, NCT05572229	Phase 2, multicentric, single arm	74	NDMM, TI	TecD	VGPR or better rate after four cycles	Recruiting	September 2030
LINKER‐MM6/EMN39, NCT06932562	Phase 3, randomized multicentric	1000	NDMM, TI	DRd induction followed by Linvo or DRd	PFS, MRD negativity rate	Recruiting	December 2036
FITFIX/EMN37, NCT07107529	Phase 2, multicentric, multiple arms	150	NDMM, TI and frail	Tec‐D, Tal‐D	PFS	Recruiting	February 2034
MajesTEC‐2, NCT04722146	Phase 1 b, multicentric, multiple arms	140	NDMM and RRMM	Tec with R, DP, DVR, DR, nirogacestat	DLTs, AEs, abnormalities in laboratory values	Active, not recruiting	October 2027
MajesTEC‐3 NCT05083169	Phase 3, randomized multicentric	587	RRMM, after one to three lines, including PI and R	Tec‐D, DVd, DPd	PFS	Active, not recruiting	December 2028
MajesTEC‐9, NCT05572515	Phase 3, randomized multicentric	614	RRMM, after one to three lines, including anti‐CD38 and R	Tec, PVd, Kd	PFS and CRS by severity	Active, not recruiting	August 2031
MonumenTAL‐6, NCT06208150	Phase 3, randomized multicentric	795	RRMM, after one to four lines, including anti‐CD38 and R	Tec‐Tal, Tal‐P, EloPd, PVd	PFS	Recruiting	March 2028
TRIMM‐2, NCT04108195	Phase 1b, multicentric	290	RRMM, after three or more lines, including PI and IMIDs	Tec‐D, Tal‐D, Tec‐DP, or Tal‐DP	DLTs, AEs	Active, not recruiting	April 2027
TRIMM‐3, NCT05338775	Phase 1b, multicentric, multiple arms	74	RRMM	Tec + PD‐1 inhibitor, Tal + PD1 inhibitor	DLTs, AEs, abnormalities in laboratory values	Active, not recruiting	May 2027
RedirecTT‐1, NCT04586426	Phase 1b‐2, multicentric	228	RRMM, TCE	Tec‐Tal	DLTs, AEs, ORR	Active, not recruiting	October 2026
NCT07105059	Phase 1, multicentric, single arm	18	RRMM, after two or more lines and TCE	Tec‐mezigdomide	DLTs	Recruiting	August 2028
NCT07463807	Phase 1b/2, multiple arms	162	RRMM, after one or two lines, including anti‐CD38 and R	Tec‐P, KPd	DLTs, MRD negativity rate	Not yet recruiting	August 2026
MagnetisMM‐5, NCT05020236	Phase 3, randomized multicentric	944	RRMM, exposed to R	Elra, Elra‐D, DPd	DLTs, AEs, PFS	Recruiting	May 2027
MagnetisMM‐20, NCT05675449	Phase 1b, multicentric, multiple arms	59	RRMM	Elra‐Kd, Elra‐maplirpacept	DLTs	Active, not recruiting	February 2029
MagnetisMM‐30, NCT06215118	Phase 1b, multicentric, single arm	87	RRMM, exposed to PI and IMID	Elra‐iberdomide	DLTs, AEs	Recruiting	March 2028
MagnetisMM‐32, NCT06152575	Phase 3, randomized multicentric	492	RRM, after one to four lines, including anti‐CD38 and R	Elra, EloPd, PVd, Kd	PFS	Recruiting	December 2027
MELT‐MM, NCT06645678	Phase 1/2, multicentric, single arm	75	RRMM after two or more lines, including PI and R	Elra‐mezigdomide	ORR	Recruiting	October 2027
NCT05927571	Phase 1b, multicentric, single arm	120	RRMM	Elra‐cevostamab	AEs, recommended phase 2 regimen	Recruiting	July 2027
NCT07280013	Phase 1b, multicentric, single arm	60	RRMM, IMID exposed	Elra‐cemisidomide	DLTs, AEs	Recruiting	June 2029
LINKER‐MM3, NCT05730036	Phase 3, randomized multicentric	410	RRMM, after one to four lines, including PI and R	Linvo, EloPd	PFS	Recruiting	April 2033
LINKER‐MM5, NCT07222761	Phase 3, randomized multicentric	915	RRMM, after one to three lines, including R and either a PI or an anti‐CD38	Linvo, Linvo‐K, SOC	PFS, MRD negativity rate, AEs	Recruiting	September 2034
NCT05137054	Phase 1b, multicentric	317	RRMM	Linvo with D, K, V, R, P, isatuximab, fianlimab, cemiplimab, nirogacestat, cevostamab	DLTs, AEs	Recruiting	September 2034
NCT07455851	Phase 1/2, multiple arms	150	RRMM, after three or more lines	Linvo‐REGN17372, Linvo^a^	DLTs, AEs, ORR, and VGPR rate	Recruiting	September 2033
COSTIMM, NCT06669247	Phase 1/2, multicentric, multiple arms	186	RRMM, after three or more lines and TCE	Linvo‐REGN7945, Linvo^b^	DLTs, AEs, ORR, and VGPR or better rate	Recruiting	November 2035

Abbreviations: AEs, adverse events; CR, complete response; d, dexamethasone; D, daratumumab; DLTs, dose limiting toxicities; Elo, elotuzumab; EloPd, elotuzumab plus pomalidomide‐dexamethasone; Elra, elranatamab; IMIDs, immunomodulatory drugs; K, carfilzomib; Linvo, linvoseltamab; MRD, measurable residual disease; NCT, ClinicalTrials.gov identifier; NDMM, newly diagnosed multiple myeloma; ORR, overall response rate; P, pomalidomide; PFS, progression‐free survival; PI, proteasome inhibitor; PVd, pomalidomide, bortezomib, and dexamethasone; R, lenalidomide; RRMM, relapsed/refractory multiple myeloma; SOC, standard of care; Tal, talquetamab; TCE, triple‐class exposed; TE, transplant‐eligible; Tec, teclistamab; TI, transplant‐ineligible; V, bortezomib; VGPR, very good partial response.

^a^ REGN17372 is an anti‐GPRC5D (G‐protein–coupled receptor family C, group 5, member D) and anti‐CD28 costimulatory bispecific antibody.

^b^ REGN7945 is an anti‐CD38 and anti‐CD28 bispecific antibody.

**TABLE 4:** Data available from ongoing clinical trials of bispecific antibodies in combination or in earlier lines.

										All grades/grade 3 and 4, %			
Trial (reference)	No. of patients	mFU, months	Setting	mLoT, [range]	Treatment	ORR, %	≥VGPR, %	MRD‐negative, %	mPFS, months	CRS	Neutropenia	Infections	No. of grade 5 infections
MajesTEC‐4/EMN30 (Zamagni 2024^120^)	94	14.4, cohort 1; 5.0, cohort 2; 4.9, cohort 3	NDMM, TE	—	R, TecR, Tec maintenance	NA	NA	100.0	NA	43.6/0.0	69.0/66.0, cohort 1; 44.0/41.0, cohort 2; 37.0/28.0, cohort 3	78.0/NA, cohort 1; 63.0/NA, cohort 2; 61.0/NA, cohort 3	1
GMMG‐HD10/DSMM‐XX, MajesTEC‐5 (Raab 2026^121^)	49	12.3	NDMM, TE	—	Tec‐DRd, Tec‐DVRd	100.0	94.0	98.0	12‐month PFS, 100.0%	67.3/0.0	65.3/59.2	81.6/36.7	0
ImmunoPLANT (Kazandjian 2025^122^)	19	3.9	NDMM, TE and TI	—	Linvo consolidation for four to six cycles	NA	NA	100.0	NA	0.0/0.0^a^	NA/5.0	NA/5.0	0
MajesTEC‐7 (Touzeau 2024^123^)	26 with Tec‐DR	10.2	NDMM, TI	—	Tec‐DR, DRd	92.3	92.3	NA	NA	61.5/0.0	NA/50.0	96.2/30.8	1
IFM 2021‐01/TecLille (Manier 2025^124^)	37	7.6	NDMM, TI	—	Tec‐D	100.0	97.0	100.0	NR	NA	NA/27.0	NA/14.0	0
MajesTEC‐2 (Searle 2023^125^)	32	5.78	RRMM	2 [1–3]	Tec‐DR	100.0	NA	NA	NA	81.3/0.0	75.0/68.8	75.0/28.1	NA
MajesTEC‐2 (Tan 2023^126^)	31	9.9	RRMM	4 [2–9]	Tec‐R	74.2	NA	NA	NA	67.7/3.0	74.2/67.7	80.6/45.2	NA
MajesTEC‐2 and TRIMM‐2 (D’Souza 2024^127^)	27	25.8	RRMM	2 [>3 in 33%]	Tec‐DP	88.5	84.6	NA	26.5	55.6/0.0	77.8/77.8	92.6/63.0	6
TRIMM‐2 (Rodriguez Otero 2022^128^)	46	7.2	RRMM	6 [2–17]	Tec‐D	78.0	73.0	NA	NA	61.0/0.0	54.0/50.0	63.0/28.0	NA
MajesTEC‐3 (Costa 2026,^111^ Banerjee, 2026^125^)	587	34.5	RRMM	2 [1–3]	Tec‐D, DPd, DVd	89.0	86.6	58.4	36‐month PFS, 83.4%	60.1/0.0	78.4/75.6	96.5/54.1	13
MajesTEC‐9 (Touzeau 2026^112^)	574	17.3	RRMM	2 [1–3]	Tec, PVd, Kd	84.5	80.1	86.4	18‐month PFS, 69.8%	66.0/0.7	62.5/54.3	82.8/41.6	16
MagnetisMM‐30 (Suvannasankha 2025^129^)	22	6.1	RRMM	2.5 [2–4]	Elra‐iberdomide	77.3	68.2	NA	NA	68.2/0.0	59.1/59.1	40.9/4.5	NA
MELT‐MM (Byun 2025^130^)	11	NA	RRMM	4 [2–6]	Elra‐mezigdomide	90.0	60.0	NA	NA	54.5/0.0	NA	NA	NA
RedirecTT‐1 (Ocio 2026,^110^ Cohen 2025^131^)	94	36.2	RRMM	4 [1–11]	Tec‐Tal	79.5	NA	NA	36‐month DoR, 71.1	80.9/2.1	74.5/70.2	NA/68.1	11
RedirecTT‐1, analysis in EMD population (Ocio 2026,^110^ Kumar 2026^132^)	90	12.6	RRMM	4 [1–10]	Tec‐Tal	61.4	NA	NA	21.6	78.0/0.0	72.0/62.0	79.0/31.0	5

Abbreviations: CRS, cytokine‐release syndrome; D, daratumumab; d, dexamethasone; DoR, duration of response; EMD, extramedullary disease; K, carfilzomib; mFU, median follow‐up; mLoT, median lines of treatment; MRD, measurable residual disease; NA, not available; NDMM, newly diagnosed multiple myeloma; NE, not estimable; NR, not reached; ORR, overall response rate; P, pomalidomide; PFS, progression free survival; R, lenalidomide; RRMM, relapsed/refractory multiple myeloma; Tal, talquetamab; TE, transplant eligible; Tec, teclistamab; TI, transplant ineligible; V, bortezomib; VGPR, very good partial response.

^a^ CRS prophylaxis with tocilizumab administered.

Regarding newly diagnosed, transplantation‐eligible patients, both teclistamab and linvoseltamab, either after or combined with standard induction therapy, demonstrated unprecedented efficacy in reaching MRD negativity.^120^, ^121^, ^122^ In transplantation‐ineligible patients, frontline therapy with teclistamab and daratumumab or daratumumab and lenalidomide is extremely effective, with ORRs >90%.^123^, ^124^ AEs in both populations were manageable, with immunoglobulin supplementation strongly limiting serious infectious complications^123^, ^124^ and less frequent teclistamab dosing also associated with less frequent AEs.^120^

In the relapsed/refractory MM setting, in the phase 3 MajesTEC‐9 study, teclistamab monotherapy showed a marked PFS and OS benefit compared with pomalidomide‐bortezomib‐dexamethasone or carfilzomib‐dexamethasone in patients who received from one to three prior lines of therapy, with CRS and ICANS rates in line with previous reports and a 41.6% rate of grade 3 or greater infection, although grade 3 or greater infections decreased over time.^112^

Concerning combination therapy, in the MajesTEC‐3 trial (ClinicalTrials.gov identifier NCT05083169), teclistamab and daratumumab were associated with a significantly lower risk of progression and death compared with daratumumab‐pomalidomide‐dexamethasone or daratumumab‐bortezomib‐dexamethasone in patients who had received from one to three prior lines of therapy, both in the whole population and in high‐risk subgroups (e.g., patients with high‐risk cytogenetic features, International Staging System stage III disease, or functional high‐risk). This also held true for patients who had prior anti‐CD38 exposure, which however only made up 5.3% of the whole study population: as anti‐CD38 exposure becomes increasingly common in these patients, real‐world studies could be useful in confirming the effectiveness of the combination in this population. Regarding safety, any‐grade, grade 3 or greater, and grade 5 infections were reported in 96.5%, 54.1%, and 4.6% of patients in the study arm, respectively, and in 84.1%, 43.4%, and 1.4% of patients in the control arm, respectively, suggesting that infectious risk remains higher with teclistamab. However, less frequent teclistamab dosing and immunoglobulin replacement limited the infectious risk: new‐onset infections sharply declined at the 6‐month mark with the passage to a monthly schedule, and fatal events mostly occurred in patients who had not received immunoglobulin support.^111^, ^133^ Teclistamab and daratumumab also showed encouraging efficacy (ORR, 78%) in the TRIMM‐2 trial (ClinicalTrials.gov identifier NCT04108195), despite a smaller population of more heavily pretreated patients (median, six prior lines) and shorter follow‐up. Grade 3 or greater infections were recorded in <30% of patients, and there were no grade 3 or greater episodes of CRS.^128^ Teclistamab with either lenalidomide or daratumumab plus lenalidomide in the MajesTEC‐2 study (ClinicalTrials.gov identifier NCT04722146) reached a 74.2% ORR in lenalidomide‐exposed patients (41.9% lenalidomide‐refractory).^125^, ^126^ Teclistamab with daratumumab and pomalidomide, in the MajesTEC‐2 and TRIMM‐2 studies, produced a promising ORR of 88.5%; it was also noted that four of six grade 5 infections occurred while the patients had a low IgG count; conversely, no grade 5 infections were observed after the implementation of more intensive antimicrobial prophylaxis.^127^ Elranatamab with cereblon E3 ligase modulators, such as mezigdomide and iberdomide, exhibited promising efficacy, with no particular safety concerns, although available data are limited.^129^, ^130^ Finally, teclistamab has been paired with talquetamab in the RedirecTT‐1 trial (ClinicalTrials.gov identifier NCT04586426), exhibiting unprecedented efficacy in a heavily pretreated population and especially in patients with EMD: the most recent data indicate an ORR of 79.5% (61.1% in patients with EMD) and a 36‐month PFS rate of 57.9% (39.7% in patients with EMD) at the recommended phase 2 dose. The infectious risk was comparable to that of bsAb monotherapy, with 54.5% of patients experiencing grade 3 or greater infections at the recommended phase 2 dose according to the most recent follow‐up, whereas previous data had described all‐grade infections in 89% of patients and grade 3 or greater infections in 64% of patients, once again with a higher incidence in the first 6 months of therapy.^110^, ^131^, ^132^

### Novel anti‐BCMA bsAbs

Alongside combining approved bsAbs with other agents, another active avenue of research is the development of novel anti‐BCMA bsAbs (active trials are listed in Table 5, and available results are detailed in Table 6).^134^, ^135^, ^136^, ^137^, ^138^, ^139^, ^140^, ^141^

**TABLE 5:** Ongoing trials with novel anti–B‐cell maturation antigen bispecific antibodies.

Trial	Study design	Estimated no. of patients	Key inclusion and exclusion criteria	Treatment regimens	Primary end points	Status	Estimated completion date
NCT06896916	Phase 1/2, multiple arms, multicentric	135	NDMM, TI	Etentamig‐iberdomide	DLTs, AEs	Recruiting	March 2036
NCT07095452	Phase 2/3, multiple arms, multicentric	660	NDMM, TI	Etentamig‐D, DRd	AEs, response rates, CRS	Recruiting	January 2042
NCT03933735	Phase 1, multiple arms, multicentric	220	RRMM, after three or more lines, TCE	Etentamig	DLTs, PK, antidrug antibodies	Active, not recruiting	May 2026
NCT06223516	Phase 1b, multiple arms, multicentric	60	RRMM, after three to five lines, TCE	Etentamig	CRS and ICANS rates, PK	Active, not recruiting	December 2027
NCT05650632	Phase 1b, multiple arms, multicentric	210	RRMM, after two or more lines, TCE	Etentamig	CRS rates, AEs	Recruiting	August 2029
NCT05259839	Phase 1, multiple arms, multicentric	283	RRMM	Etentamig‐Rd, etentamib‐Dd, etentamig‐Pd	DLTs, AEs	Active, not recruiting	September 2033
NCT06892522	Phase 1/2, multiple arms, multicentric	440	RRMM	Etentamig, etentamig‐DR, etentamig‐R, etentamig‐Kd, DRd, R, DKd	DLTs, AEs	Recruiting	March 2036
CERVINO NCT06158841	Phase 3, randomized multicentric	380	RRMM, after three or more lines, TCE	Etentamig, Kd, EloPd, SVd	PFS, ORR	Recruiting	December 2027
NCT06574568	Phase 1, single arm, multicentric	70	RRMM, exposed to at least two among PIs, IMIDs, and anti‐CD38 agents	YKST02	DLTs, ORR	Recruiting	June 2027
CAREMM‐007 NCT07409454	Phase 2, single arm, no data on locations	20	NDMM, TE	CM336 maintenance	MRD negativity conversion rate	Not yet recruiting	December 2027
NCT05299424	Phase 1/2, multiple arms, multicentric	48	RRMM, after two or more lines	CM336	DLTs, AEs, ORR	Recruiting	December 2026
NCT07407010	Phase 1, monocentric, single arm	10	RRMM	CM336 as bridge to CAR T‐cell therapy	ORR, AEs	Active, not recruiting	May 2028

Abbreviations: AEs, adverse events; CAR T‐cell therapy, chimeric antigen receptor T‐cell cell therapy; CRS, cytokine‐release syndrome; D, daratumumab; d, dexamethasone; DKd, daratumumab, carfilzomib, and dexamethasone; DLTs, dose‐limiting toxicities; DR, daratumumab and lenalidomide; DRd, daratumumab, lenalidomide, and dexamethasone; EloPd, elotuzumab plus pomalidomide‐dexamethasone; ICANS, immune effector cell–associated neurotoxicity syndrome; IMIDs, immunomodulatory drugs; K, carfilzomib; Kd, carfilzomib and dexamethasone; MRD, measurable residual disease; NDMM, newly diagnosed multiple myeloma; ORR, overall response rate; P, pomalidomide; Pd, pomalidomide and dexamethasone; PFS, progression‐free survival; PIs, proteasome inhibitors; PK, pharmacokinetics; R, lenalidomide; Rd, lenalidomide and dexamethasone; RRMM, relapsed/refractory multiple myeloma; SVd, selinexor, velcade and dexamethasone; TCE, triple‐class exposed; TE, transplant‐eligible; TI, transplant‐ineligible; V, bortezomib.

**TABLE 6:** Available data from clinical trials with novel anti–B‐cell maturation antigen bispecific antibodies.

											All grades/grade 3 and 4, %			
Trial (reference)	No. of patients	mFU, months	Setting	mLoT, [range], no.	Exposure/refractoriness to previous therapies, %	Treatment	ORR, %	≥VGPR, %	MRD‐negative, %	mPFS or DoR, months	CRS	Neutropenia	Infections	No. of grade 5 infections
NCT03287908 (Harrison 2020^134^)	75	6.1, mDoT; 1.7, on treatment	RRMM	6 [1–25]	68.0 TCR	AMG‐701	36.0	22.0	4.0	mDoR, 3.8	61.0/7.0	23.0/NA	NA/17.0	2
NCT03486067 (Bar 2026^135^)	95	11.8	RRMM	4 [3–14]	64.2 TCR, 20.0 penta‐refractory	Alnuctamab sc	58.9	46.3	49.5	mPFS, 11.5	57.9/0	53.7/43.2	64.2/14.7	1
NCT04083534 (Suvannasankha 2023^136^)	43	7	RRMM	5 [2–9]	100.0 TCE, 61.0 TCR	REGN‐5459	67.0	58.0	NA	12‐month DoR, 66.7%	54.0/5.0	37.0/37.0	61.0/37.0	2
NCT05299424 (An 2024^137^)	25	8	RRMM	4 [2–15]	100.0 TCE	CM‐336	60.9	NA	88.9; nine patients in sCR	NR	68.0/4.0	56.0/44.0	NA/NA	NA
NCT03933735 and NCT05650632 (Baljevic 2025^138^)	146	13	RRMM	4 [3–23]	NA	Etentamig	66.0	54.0	NA	12‐month PFS estimate, 55.0%	NA	NA/38.0	NA/22.0	NA
NCT03933735 (Rodriguez Valdes 2024^139^)	220	4.1, cohort 1; 12.2, cohort 2; 24.2, cohort 3	RRMM	5 [3–23]	100.0 TCE	Etentamig 60 mg Q4W, cohort 1; 40 mg Q3W. cohort 2; 60 mg Q3W, cohort 3	65.0, cohort 1; 64.0, cohort 2; 60.0, cohort 3	50.0, cohort 1; 53.0, cohort 2; 52.0, cohort 3	NA	NA	43.0/0.0, cohort 1; 71.0/0.0, cohort 2; 70.0/2.0, cohort 3	NA/14.0, cohort 1; NA/31.0, cohort 2; NA/31.0, cohort 3	NA/10.0, cohort 1; NA/24.0, cohort 2; NA/34.0, cohort 3	NA
NCT05259839 (Rodriguez 2024^140^)	74	5	RRMM	4 [3–9]	57.0 anti–CD38‐refractory	Etentamig‐Dd	70.0	NA	NA	NR	27.0/4.0	39.0/38.0	NA/NA	NA
NCT05259839 (Voorhees 2025^141^)	85	18	RRMM	4 [2‐10]	73.0 TCR, 49.0 Pom‐refractory	Etentamig‐Pd	81.0/72.0		NA	12‐month PFS estimate, 69.6%	37.0/0.0	NA/78.0	NA/49.0	1

Abbreviations: D, daratumumab; DoR, duration of response; Etentamig‐Dd, etentamig plus daratumumab‐dexamethasone; Etentamig‐Pd, etentamig plus pomalidomide‐dexamethasone; exp/ref, exposed/refractory; mDoR, median duration of response; mDoT, median duration of treatment; mFU, median follow‐up; mLoT, median lines of treatment; mPFS, median progression‐free survival; MRD, measurable residual disease; NA, not available; NCT, ClinicalTrials.gov identifier; NE, not estimable; ORR, overall response rate; Penta, treatment with proteasome inhibitors, immunomodulatory drugs, and anti‐CD38 monoclonal antibodies; PFS, progression‐free survival; Pom, pomalidomide; Q3W, every 3 weeks; Q4W, every 4 weeks; RRMM, relapsed/refractory multiple myeloma; sc, subcutaneous; sCR, stringent complete response; TCE, triple‐class exposed; TCR, triple‐class refractory; VGPR, very good partial response.

The first‐in‐class anti‐BCMA bsAb to demonstrate efficacy in preclinical and clinical trials was AMG‐420,^142^, ^143^ although it was later discontinued because of an unfavorable pharmacokinetic profile^144^; similarly, AMG‐701^118^, ^134^, ^145^ and alnuctamab^135^, ^146^ have only reached early phases of clinical development.

Among bsAbs in active clinical development, the most advanced is etentamig, an IgG bsAb with bivalent BCMA and low‐affinity CD3 binding, translating into reduced cytokine secretion with preserved cytotoxicity.^147^ Notably, this low affinity and prolonged dosing interval (every 4 weeks) may underlie the reduced T‐cell dysfunction and infectious risk in trials.^148^ After promising results in preclinical models,^149^ monotherapy in relapsed/refractory MM produced an ORR of 66% and a 12‐month estimated PFS of 55% across two trials in TCE patients.^138^, ^139^ Combined with daratumumab and dexamethasone, etentamig produced a 70% ORR in a population, including 57% of patients who were refractory to previous anti‐CD38 therapy, although the median follow‐up was only 5 months.^140^ Combination with pomalidomide and dexamethasone in a population with 49% pomalidomide‐refractory patients achieved an 81% ORR, with the mPFS not reached in the 18‐month follow‐up.^141^ It is interesting to note that etentamig is also being studied in light‐chain amyloidosis (as detailed below).

Further bsAbs in development include REGN‐5459^136^ and, in China, CM336^137^ and YKST02. Finally, further advances in this area are expected to come from trispecific antibodies, such as ramantamig (already being compared with teclistamab in the TRIlogy‐4 [ClinicalTrials.gov identifier NCT07258511] and TRIlogy‐5 [ClinicalTrials.gov identifier NCT07518186] studies), ISB 2001, and BTL‐101 (which, after producing encouraging preclinical efficacy,^150^ is currently being tested in a phase 1/2 clinical study).

### Anti‐BCMA bsAbs in other monoclonal gammopathies and conditions

Anti‐BCMA bsAbs have also been examined as treatment for other conditions. Some studies have focused on bsAbs as a treatment strategy for smoldering MM (SMM) and for monoclonal gammopathy of undetermined significance. For high‐risk SMM, ongoing trials include: Immuno‐PRISM (ClinicalTrials.gov identifier NCT05469893), a phase 2 randomized trial comparing teclistamab with lenalidomide‐dexamethasone; REVIVE (ClinicalTrials.gov identifier NCT06100237), a phase 2 trial evaluating the rate of MRD negativity after teclistamab and daratumumab combination therapy; ERASMM (ClinicalTrials.gov identifier NCT06183489), a phase 2 study evaluating the efficacy of elranatamab; and LINKER‐SMM1 (ClinicalTrials.gov identifier NCT05955508), a phase 2 multicentric study of the safety and efficacy of linvoseltamab.

Preliminary data from the Immuno‐PRISM study are available, reporting on 45 patients who are receiving teclistamab and 14 who are receiving lenalidomide‐dexamethasone. After a median follow‐up of 23.4 months, the study reports a 87% versus 14% rate of at least a very‐good partial response (VGPR) and a 92% versus 51% 2‐year PFS rate in the teclistamab versus lenalidomide‐dexamethasone treatment arms, respectively; grade 1 and 2 CRS occurred in 71% (all grade 1–2) and grade 3 infections occurred in 20% of the patients receiving teclistamab (upon immunoglobulin supplementation in all).^151^ In the ERASMM study, 50 patients have received elranatamab to date, with an ORR of 92% and a 9‐month PFS rate of 95%; of these, 68% patients have experienced CRS, with two grade 3 events, and 50% have experienced infections, with 14% grade 3 and 4 events.^152^ Results on 24 patients from the LINKER‐SMM1 study are also available: the ORR was 100%, and 74% of patients achieved a VGPR or better. CRS was recorded in 42% of patients (all grade 1 except one grade 2), and infections were recorded in 79% (three grade 3).^153^ Finally, the phase 2 multicentric LINKER‐MGUS1 trial (ClinicalTrials.gov identifier NCT06140524) is evaluating the safety and efficacy of linvoseltamab in patients with nonhigh–risk SMM and high‐risk monoclonal gammopathy of undetermined significance.

Meanwhile, most available real‐world data come from light‐chain amyloidosis, in which cells from both preclinical and in ex‐vivo studies have been shown to express membrane‐bound and sBCMA much like MM PCs.^154^, ^155^ The first case reports on teclistamab, collecting a range from one to eight patients each and with a median follow‐up of 1–9 months, showed encouraging data, with high hematologic response rates (ranging from two third of patients in one report to 100% in most others); however, one death from cardiac involvement was also reported. A wide range of CRS incidence was reported (0%–75%), but no grade 3 or greater episodes or further complications were reported from cardiac or renal decompensation; the proportion of patients who had grade 3 or greater infections ranged from 0% to 29%.^156^, ^157^, ^158^, ^159^ A larger retrospective study, which included 17 patients with 3‐month median follow‐up, confirmed these early observations, with 88% of patients achieving a VGPR, 53% experiencing CRS (all grade 1), and 29% experiencing grade 3 or greater infections; once again, one death from organ progression was reported.^160^ Another case series of nine patients who received elranatamab and had a median follow‐up of 8.2 months reported 89% ORR and an mPFS of 8.2 months (range, 1.4–12.3 months), again with one death from organ involvement; six patients developed CRS (one grade 3 event), but no infections were registered.^161^ Finally, another study among patients who had amyloidosis treated with different bsAbs (median follow‐up, 8.8 months) reported a 100% ORR with elranatamab, a 74% ORR with teclistamab, and CRS rates of 50% (elranatamab) and 42% (teclistamab), with no grade 3 or greater events; in the whole patient cohort, two cardiac deaths were recorded.^162^ Finally, etentamig is also being developed for amyloidosis, with an active phase ½, multicentric clinical trial testing its use as monotherapy in relapsed/refractory amyloidosis (ClinicalTrials.gov identifier NCT06158854). Preliminary results in 12 patients indicated an encouraging VGPR rate of 100%, with no progressions during the median follow‐up of 9.4 months (range, 8.3–13.8 months), a 17% CRS rate (all grade 1), and only one grade 3 infection (no grade 5 events).^163^

Collectively, these data suggest that anti‐BCMA bsAbs have the potential to induce deep and fast responses, with no additional safety signals for CRS or infections, although longer follow‐up is needed to confirm these observations. However, in this pretreated and extremely frail population, amyloid‐related organ dysfunction leading to fatal events is a real risk and calls for careful patient selection.^156^, ^157^, ^158^, ^159^, ^160^, ^161^, ^162^

Information on other monoclonal gammopathies of clinical significance is scarcer: one case report describes the successful treatment of a patient who had POEMS (polyneuropathy, organomegaly, endocrinopathy, monoclonal gammopathy and skin lesions) with teclistamab, reporting a deep, rapid, and durable response that persisted 2 years after treatment initiation and no AEs of note.^164^ Another case report describes one patient who received teclistamab for cryoglobulinemia; the patient had a rapid hematologic response and certain improvement on clinical symptoms, but they had three grade 3 infections during treatment.^165^ A case series also describes five patients who received treatment with teclistamab for systemic sclerosis, with clinical improvement and reduction in antibody titers and PCs in skin biopsies; however, all patients experienced CRS, with one grade 3 event, and most had infectious complications.^166^

Finally, the field of application of bsAbs may widen even more in the future. In autoimmune diseases, CM336 is being investigated as treatment for autoimmune bullous disease (ClinicalTrials.gov identifier NCT06900010) and immune thrombocytopenia (ClinicalTrials.gov identifier NCT06799611), and REGN‐5459 is being investigated as treatment for lupus nephritis (ClinicalTrials.gov identifier NCT06975787). REGN‐5459 is also being tested in future kidney transplantation recipients who are highly sensitized to human leukocyte antigen, to lower antihuman leukocyte antigen antibodies (ClinicalTrials.gov identifier NCT05092347 and NCT05106387). Finally, an ongoing phase 1/2 clinical trial is testing etentamig monotherapy for patients with relapsed/refractory Waldestrom macroglobulinemia (ClinicalTrials.gov identifier NCT07420959).

## CONCLUSIONS

In conclusion, anti‐BCMA bsAbs have rapidly become a cornerstone of therapy for patients with TCE MM in the past few years, dramatically improving their prognosis, and these agents are set to acquire an even more important role in earlier lines of therapy in the next few years as well as potentially gaining new therapeutic indications in other diseases.

As their therapeutic indications broaden, one challenge is to better define the placement of anti‐BCMA bsAbs in the treatment algorithm of MM, especially considering the presence of anti‐BCMA CAR T cells and belantamab mafodotin: for instance, appropriate patient selection becomes of paramount importance, and more information on the efficacy of sequential anti‐BCMA therapies will be useful to choose the order of their use. In addition, to date, we have learned much about their toxicities and how to best manage them, although open questions persist, leaving space for improvement (e.g., defining the best strategy for CRS prophylaxis or immunoglobulin support or the potential safety benefits of adopting a fixed‐duration approach rather than treating until progression), which will need to be addressed promptly as their therapeutic indications widen.

## AUTHOR CONTRIBUTIONS

**Enrica Manzato**: Conceptualization; data curation; investigation; writing—original draft. **Gloria Cocchetto**: Data curation; investigation; writing—original draft. **Simona Barbato**: Conceptualization; project administration; writing—original draft; writing—review and editing. **Michele Puppi**: Investigation; writing—review and editing. **Simone Masci**: Investigation; writing—review and editing. **Elena Zamagni**: Conceptualization; data curation; supervision; funding acquisition; resources; writing—review and editing.

## CONFLICT OF INTEREST STATEMENT

Enrica Manzato reports honoraria from GlaxoSmithKline outside the submitted work. Michele Puppi reports honoraria from GlaxoSmithKline, Menarini‐Stemline, and Sanofi outside the submitted work. Elena Zamagni reports personal/consulting fees or honoraria from Amgen, AstraZeneca, Bristol Myers Squibb Company, GlaxoSmithKline, Janssen Cilag EMEA, Menarini‐Stemline, Oncopeptides AB, Pfizer, Roche, Sanofi, and Takeda Pharmaceutical Company outside the submitted work. The remaining authors disclosed no conflicts of interest.

## References

[1] Malard F , Neri P , Bahlis NJ , et al. Multiple myeloma. Nat Rev Dis Primers. 2024;10(1):45. doi:10.1038/s41572-024-00529-7 38937492

[2] Puertas B , González‐Calle V , Sobejano‐Fuertes E , et al. Novel agents as main drivers for continued improvement in survival in multiple myeloma. Cancers (Basel). 2023;15(5):1558. doi:10.3390/cancers15051558 PMC10000382 36900349

[3] Mateos MV , Weisel K , De Stefano V , et al. LocoMMotion: a study of real‐life current standards of care in triple‐class exposed patients with relapsed/refractory multiple myeloma—2‐year follow‐up (final analysis). Leukemia. 2024;38(12):2554‐2560. doi:10.1038/s41375-024-02404-6 PMC11588650 39322709

[4] Dimopoulos MA , Terpos E , Boccadoro M , et al. EHA–EMN evidence‐based guidelines for diagnosis, treatment and follow‐up of patients with multiple myeloma. Nat Rev Clin Oncol. 2025;22(9):680‐700. doi:10.1038/s41571-025-01041-x 40624367

[5] Laâbi Y , Gras M , Carbonnel F , et al. A new gene, BCM, on chromosome 16 is fused to the interleukin 2 gene by a t(4;16)(q26;p13) translocation in a malignant T cell lymphoma. EMBO J. 1992;11:3897‐3904. doi:10.1002/j.1460-2075.1992.tb05482.x PMC556899 1396583

[6] Carpenter RO , Evbuomwan MO , Pittaluga S , et al. B‐cell maturation antigen is a promising target for adoptive T‐cell therapy of multiple myeloma. Clin Cancer Res. 2013;19(8):2048‐2060. doi:10.1158/1078-0432.ccr-12-2422 PMC3630268 23344265

[7] Bu DX , Singh R , Choi EE , et al. Pre‐clinical validation of B cell maturation antigen (BCMA) as a target for T cell immunotherapy of multiple myeloma. Oncotarget. 2018;9(40):25764‐25780. doi:10.18632/oncotarget.25359 PMC5995247 29899820

[8] Zhang M , Gray F , Cushman I , et al. A novel BCMA immunohistochemistry assay reveals a heterogenous and dynamic BCMA expression profile in multiple myeloma. Mod Pathol. 2023;36(4):100050. doi:10.1016/j.modpat.2022.100050 36788077

[9] Schuh E , Musumeci A , Thaler FS , et al. Human plasmacytoid dendritic cells display and shed B cell maturation antigen upon TLR engagement. J Immunol. 2017;198(8):3081‐3088. doi:10.4049/jimmunol.1601746 28283566

[10] Alexaki VI , Pelekanou V , Notas G , et al. B‐cell maturation antigen (BCMA) activation exerts specific proinflammatory effects in normal human keratinocytes and is preferentially expressed in inflammatory skin pathologies. Endocrinology. 2012;153(2):739‐749. doi:10.1210/en.2011-1504 22166983

[11] Alexaki VI , Notas G , Pelekanou V , et al. Adipocytes as immune cells: differential expression of TWEAK, BAFF, and APRIL and their receptors (Fn14, BAFF‐R, TACI, and BCMA) at different stages of normal and pathological adipose tissue development. J Immunol. 2009;183(9):5948‐5956. doi:10.4049/jimmunol.0901186 19828625

[12] Pelekanou V , Notas G , Kampa M , et al. BAFF, APRIL, TWEAK, BCMA, TACI and Fn14 proteins are related to human glioma tumor grade: immunohistochemistry and public microarray data meta‐analysis. PLoS One. 2013;8(12):e83250. doi:10.1371/journal.pone.0083250 PMC3869762 24376672

[13] Bossen C , Schneider P . BAFF, APRIL and their receptors: structure, function and signaling. Semin Immunol. 2006;18(5):263‐275. doi:10.1016/j.smim.2006.04.006 16914324

[14] O’Connor BP , Raman VS , Erickson LD , et al. BCMA is essential for the survival of long‐lived bone marrow plasma cells. J Exp Med. 2004;199(1):91‐98. doi:10.1084/jem.20031330 PMC1887725 14707116

[15] Peperzak V , Vikström I , Walker J , et al. Mcl‐1 is essential for the survival of plasma cells. Nat Immunol. 2013;14(3):290‐297. doi:10.1038/ni.2527 PMC4041127 23377201

[16] Laurent SA , Hoffmann FS , Kuhn PH , et al. γ‐Secretase directly sheds the survival receptor BCMA from plasma cells. Nat Commun. 2015;6(1):7333. doi:10.1038/ncomms8333 PMC4490565 26065893

[17] Sanchez E , Gillespie A , Tang G , et al. Soluble B‐cell maturation antigen mediates tumor‐induced immune deficiency in multiple myeloma. Clin Cancer Res. 2016;22(13):3383‐3397. doi:10.1158/1078-0432.ccr-15-2224 26960399

[18] Novak AJ , Darce JR , Arendt BK , et al. Expression of BCMA, TACI, and BAFF‐R in multiple myeloma: a mechanism for growth and survival. Blood. 2004;103(2):689‐694. doi:10.1182/blood-2003-06-2043 14512299

[19] Moreaux J , Legouffe E , Jourdan E , et al. BAFF and APRIL protect myeloma cells from apoptosis induced by interleukin 6 deprivation and dexamethasone. Blood. 2004;103(8):3148‐3157. doi:10.1182/blood-2003-06-1984 PMC2387243 15070697

[20] Tai YT , Li XF , Breitkreutz I , et al. Role of B‐cell–activating factor in adhesion and growth of human multiple myeloma cells in the bone marrow microenvironment. Cancer Res. 2006;66(13):6675‐6682. doi:10.1158/0008-5472.can-06-0190 16818641

[21] Tai YT , Acharya C , An G , et al. APRIL and BCMA promote human multiple myeloma growth and immunosuppression in the bone marrow microenvironment. Blood. 2016;127(25):3225‐3236. doi:10.1182/blood-2016-01-691162 PMC4920023 27127303

[22] Salem DA , Maric I , Yuan CM , et al. Quantification of B‐cell maturation antigen, a target for novel chimeric antigen receptor T‐cell therapy in myeloma. Leuk Res. 2018;71:106‐111. doi:10.1016/j.leukres.2018.07.015 PMC8063571 30053652

[23] Dogan A , Siegel D , Tran N , et al. B‐cell maturation antigen expression across hematologic cancers: a systematic literature review. Blood Cancer J. 2020;10(6):73. doi:10.1038/s41408-020-0337-y PMC7327051 32606424

[24] Sanchez E , Li M , Kitto A , et al. Serum B‐cell maturation antigen is elevated in multiple myeloma and correlates with disease status and survival. Br J Haematol. 2012;158(6):727‐738. doi:10.1111/j.1365-2141.2012.09241.x 22804669

[25] Costa BA , Ortiz RJ , Lesokhin AM , Richter J . Soluble B‐cell maturation antigen in multiple myeloma. Am J Hematol. 2024;99(4):727‐738. doi:10.1002/ajh.27225 38270277

[26] Labrijn AF , Janmaat ML , Reichert JM , Parren PWHI . Bispecific antibodies: a mechanistic review of the pipeline. Nat Rev Drug Discov. 2019;18(8):585‐608. doi:10.1038/s41573-019-0028-1 31175342

[27] Ellerman D . Bispecific T‐cell engagers: towards understanding variables influencing the in vitro potency and tumor selectivity and their modulation to enhance their efficacy and safety. Methods. 2019;154:102‐117. doi:10.1016/j.ymeth.2018.10.026 30395966

[28] Friedrich MJ , Neri P , Kehl N , et al. The pre‐existing T cell landscape determines the response to bispecific T cell engagers in multiple myeloma patients. Cancer Cell. 2023;41(4):711‐725.e6. doi:10.1016/j.ccell.2023.02.008 36898378

[29] Panowski SH , Kuo TC , Zhang Y , et al. Preclinical efficacy and safety comparison of CD3 bispecific and ADC modalities targeting BCMA for the treatment of multiple myeloma. Mol Cancer Ther. 2019;18(11):2008‐2020. doi:10.1158/1535-7163.mct-19-0007 31434693

[30] Pillarisetti K , Powers G , Luistro L , et al. Teclistamab is an active T cell–redirecting bispecific antibody against B‐cell maturation antigen for multiple myeloma. Blood Adv. 2020;4(18):4538‐4549. doi:10.1182/bloodadvances.2020002393 PMC7509877 32956453

[31] DiLillo DJ , Olson K , Mohrs K , et al. A BCMAxCD3 bispecific T cell–engaging antibody demonstrates robust antitumor efficacy similar to that of anti‐BCMA CAR T cells. Blood Adv. 2021;5:1291‐1304. doi:10.1182/bloodadvances.2020002736 PMC7948265 33651100

[32] Brinkmann U , Kontermann RE . The making of bispecific antibodies. mAbs. 2017;9(2):182‐212. doi:10.1080/19420862.2016.1268307 PMC5297537 28071970

[33] Liu H , Saxena A , Sidhu SS , Wu D . Fc engineering for developing therapeutic bispecific antibodies and novel scaffolds. Front Immunol. 2017;8:38. doi:10.3389/fimmu.2017.00038 PMC5266686 28184223

[34] Rees MJ , Mammadzadeh A , Bolarinwa A , et al. Clinical features associated with poor response and early relapse following BCMA‐directed therapies in multiple myeloma. Blood Cancer J. 2024;14(1):122. doi:10.1038/s41408-024-01081-z PMC11266661 39043638

[35] Moreau P , Garfall AL , van de Donk NWCJ , et al. Teclistamab in relapsed or refractory multiple myeloma. N Engl J Med. 2022;387(6):495‐505. doi:10.1056/NEJMoa2203478 PMC10587778 35661166

[36] Lesokhin AM , Tomasson MH , Arnulf B , et al. Elranatamab in relapsed or refractory multiple myeloma: phase 2 MagnetisMM‐3 trial results. Nat Med. 2023;29(9):2259‐2267. doi:10.1038/s41591-023-02528-9 PMC10504075 37582952

[37] Lee H , Durante M , Skerget S , et al. Impact of soluble BCMA and non–T‐cell factors on refractoriness to BCMA‐targeting T‐cell engagers in multiple myeloma. Blood. 2024;144(25):2637‐2651. doi:10.1182/blood.2024026212 PMC11738017 39321344

[38] van de Donk N , Cortes‐Selva D , Casneuf T , et al. P32 MajesTEC‐1: correlative analyses of teclistamab, a B‐cell maturation antigen (BCMA) x CD3 bispecific antibody, in patients with relapsed/refractory multiple myeloma (RRMM) [abstract]. Hemasphere. 2023;7(S2):28‐29. doi:10.1097/01.hs9.0000936256.84340.a7

[39] Truger MS , Duell J , Zhou X , et al. Single‐ and double‐hit events in genes encoding immune targets before and after T cell–engaging antibody therapy in MM. Blood Adv. 2021;5(19):3794‐3798. doi:10.1182/bloodadvances.2021004418 PMC8679680 34471932

[40] Papadimitriou M , Ahn S , Diamond BT , et al. Timing genomic antigen loss in multiple myeloma treated with T‐cell redirecting immunotherapies. Blood Cancer Discov. 2025;6:572‐579. doi:10.1158/2643-3230.bcd-25-0005 PMC12514755 40906971

[41] Lee H , Ahn S , Vishwamitra D , et al. Antigen‐dependent clonal sweep with sequential or combinatory anti‐myeloma T cell engagers [abstract]. Blood. 2025;146(suppl 1):1030. doi:10.1182/blood-2025-1030

[42] Lee H , Ahn S , Maity R , et al. Mechanisms of antigen escape from BCMA‐ or GPRC5D‐targeted immunotherapies in multiple myeloma. Nat Med. 2023;29(9):2295‐2306. doi:10.1038/s41591-023-02491-5 PMC10504087 37653344

[43] Lee H , Ahn S , Poorebrahim M , et al. Tumor intrinsic and antigen‐independent resistance mechanisms to bispecific T cell engagers in multiple myeloma [abstract]. Blood. 2024;144(suppl 1):1892. doi:10.1182/blood-2024-211979

[44] Chen H , Li M , Xu N , et al. Serum B‐cell maturation antigen (BCMA) reduces binding of anti‐BCMA antibody to multiple myeloma cells. Leuk Res. 2019;81:62‐66. doi:10.1016/j.leukres.2019.04.008 31035033

[45] Chen H , Yu T , Lin L , et al. γ‐Secretase inhibitors augment efficacy of BCMA‐targeting bispecific antibodies against multiple myeloma cells without impairing T‐cell activation and differentiation. Blood Cancer J. 2022;12(8):118. doi:10.1038/s41408-022-00716-3 PMC9381512 35973981

[46] Meermeier EW , Welsh SJ , Sharik ME , et al. Tumor burden limits bispecific antibody efficacy through T‐cell exhaustion averted by concurrent cytotoxic therapy. Blood Cancer Discov. 2021;2(4):354‐369. doi:10.1158/2643-3230.bcd-21-0038 PMC8266040 34258584

[47] Chalopin T , Cellerin E , Demarquette H , et al. Debulking strategy prior to anti‐BCMA/CD3 bispecific antibodies in extramedullary and/or high tumor burden RRMM: a retrospective cohort study. Blood Cancer J. 2025;15(1):174. doi:10.1038/s41408-025-01365-y PMC12540854 41120261

[48] Philipp N , Kazerani M , Nicholis A , et al. T‐cell exhaustion induced by continuous bispecific molecule exposure is ameliorated by treatment‐free intervals. Blood. 2022;140(10):1104‐1118. doi:10.1182/blood.2022015956 PMC10652962 35878001

[49] European Medicines Agency (EMA) . Summary of Product Characteristics: Elranatamab [EMA package insert]. EMA; 2023. Accessed June 15, 2026. https://www.ema.europa.eu/en/documents/product‐information/elrexfio‐epar‐product‐information_en.pdf

[50] European Medicines Agency (EMA) . Summary of Product Characteristics: Teclistamab [EMA package insert; Italian]. EMA; 2026. Accessed June 15, 2026. https://ec.europa.eu/health/documents/community‐register/2022/20220823156764/anx_156764_it.pdf

[51] European Medicines Agency (EMA) . Summary of Product Characteristics: Linvoseltamab [EMA package insert]. EMA; 2025. Accessed June 15, 2026. https://www.ema.europa.eu/en/documents/product‐information/lynozyfic‐epar‐product‐information_en.pdf

[52] US Food and Drug Administration (FDA) . Highlights of Prescribing Information: Teclistamab. FDA; 2026. Accessed June 15, 2026. https://www.accessdata.fda.gov/drugsatfda_docs/label/2026/761291s018lbl.pdf

[53] US Food and Drug Administration (FDA) . Highlights of Prescribing Information: Elranatamab. FDA; 2026. Accessed June 15, 2026. https://www.accessdata.fda.gov/drugsatfda_docs/label/2026/761345s007lbl.pdf

[54] US Food and Drug Administration (FDA) . Highlights of Prescribing Information: Linvoseltamab. FDA; 2025. Accessed June 15, 2026. https://www.accessdata.fda.gov/drugsatfda_docs/label/2025/761400s000lbl.pdf

[55] Garfall AL , Nooka AK , van de Donk NWCJ , et al. Long‐term follow‐up from the phase 1/2 MajesTEC‐1 trial of teclistamab in patients with relapsed/refractory multiple myeloma [abstract]. J Clin Oncol. 2024;42(16 suppl):7540. doi:10.1200/JCO.2024.42.16_suppl.7540

[56] Bahlis NJ , Costello CL , Raje NS , et al. Elranatamab in relapsed or refractory multiple myeloma: the MagnetisMM‐1 phase 1 trial. Nat Med. 2023;29(10):2570‐2576. doi:10.1038/s41591-023-02589-w PMC10579053 37783970

[57] Prince HM , Bahlis NJ , Rodíguez‐Ortero , et al. 654. MagnetisMM‐3: long‐term update and efficacy and safety of less frequent dosing of elranatamab in patients with relapsed or refractory multiple myeloma [abstract]. Blood. 2024;144(suppl 1):4738‐4739. doi:10.1182/blood-2024-208192

[58] Tomasson MH , Iida S , Niesvizky R , et al. Long‐term survival and safety of elranatamab in patients with relapsed or refractory multiple myeloma: update from the MagnetisMM‐3 study [letter]. Hemasphere. 2024;8(7):e136. doi:10.1002/hem3.136 PMC11269363 39055646

[59] Bumma N , Richter J , Jagannath S , et al. Linvoseltamab for treatment of relapsed/refractory multiple myeloma. J Clin Oncol. 2024;42(22):2702‐2712. doi:10.1200/jco.24.01008 PMC11272139 38879802

[60] Lee HC , Zonder JA , Dhodapkar MV , et al. Linvoseltamab in patients with relapsed/refractory multiple myeloma in the LINKER‐MM1 study: longer follow‐up and subgroup analyses. Clin Lymphoma Myeloma Leuk. 2026;26(2):e201‐e212.e8. doi:10.1016/j.clml.2025.11.004 41387038

[61] Uttervall K , Kortum MK , Perrot A , et al. REALiTEC: a multi‐country observational retrospective study of teclistamab in patients with relapsed/refractory multiple myeloma outside of clinical trials. Haematologica. 2026;111(9):3055‐3065. doi:10.3324/haematol.2025.289281 PMC13530970 41784028

[62] Tan CR , Asoori S , Huang CY , et al. Real‐world evaluation of teclistamab for the treatment of relapsed/refractory multiple myeloma (RRMM): an International Myeloma Working Group study. Blood Cancer J. 2025;15(1):53. doi:10.1038/s41408-025-01259-z PMC11965530 40175336

[63] Riedhammer C , Bassermann F , Besemer B , et al. Real‐world analysis of teclistamab in 123 RRMM patients from Germany. Leukemia. 2024;38(2):365‐371. doi:10.1038/s41375-024-02154-5 PMC10844072 38245601

[64] Razzo BM , Midha S , Portuguese AJ , et al. Real‐world experience with teclistamab for relapsed/refractory multiple myeloma from the US Myeloma Immunotherapy Consortium. Blood Cancer Discov. 2025;6:561‐571. doi:10.1158/2643-3230.bcd-24-0354 40629516

[65] Perrot A , Hulin C , Boumendil A , et al. Teclistamab in relapsed refractory multiple myeloma: a multi‐institutional real‐world study from the French early access program. Haematologica. 2024;110(4):990‐994. doi:10.3324/haematol.2024.286118 PMC11959243 39568438

[66] Mohan M , Monge J , Shah N , et al. Teclistamab in relapsed refractory multiple myeloma: multi‐institutional real‐world study. Blood Cancer J. 2024;14(1):35. doi:10.1038/s41408-024-01003-z PMC10914756 38443345

[67] Dima D , Davis JA , Ahmed N , et al. Safety and efficacy of teclistamab in patients with relapsed/refractory multiple myeloma: a real‐world experience. Transplant Cell Ther. 2024;30(3):308.e1‐308.e13. doi:10.1016/j.jtct.2023.12.016 38151105

[68] Dhakal B , Levy MY , Ko G , et al. Real‐world patient characteristics, treatment patterns, and clinical outcomes in patients with relapsed or refractory multiple myeloma receiving teclistamab: a panel chart review study. Oncol Ther. 2026;14(2):671‐691. doi:10.1007/s40487-026-00429-8 PMC13304050 41886245

[69] Portuguese AJ , Davis JA , Raza S , et al. Real‐world outcomes with elranatamab in multiple myeloma: a multicenter analysis from the U.S. Multiple Myeloma Immunotherapy Consortium. Blood Cancer J. 2026;16(1):47. doi:10.1038/s41408-026-01477-z PMC13039266 41896552

[70] Leleu X , Bahlis N , Rodriguez‐Otero P , et al. P‐286 Impact of hypogammaglobulinemia and immunoglobulin replacement therapy on infection rate in patients with RRMM treated with elranatamab: post‐hoc analysis from MagnetisMM‐3 [abstract]. Clin Lymphoma Myeloma Leuk. 2023;23:S194. doi:10.1016/s2152-2650(23)01904-3

[71] Krishnan A , Nooka AK , Chari A , et al. Teclistamab versus real‐world physician’s choice of therapy in triple‐class exposed relapsed/refractory multiple myeloma. J Comp Eff Res. 2023;12(6):e220186. doi:10.57264/cer-2022-0186 PMC10402759 37114426

[72] Mateos M‐V , Chari A , Usmani SZ , et al. Comparative efficacy of teclistamab versus physician’s choice of therapy in the long‐term follow‐up of APOLLO, POLLUX, CASTOR, and EQUULEUS clinical trials in patients with triple‐class exposed relapsed or refractory multiple myeloma. Clin Lymphoma Myeloma Leuk. 2023;23(5):385‐393. doi:10.1016/j.clml.2023.02.006 36967244

[73] Moreau P , Mateos MV , Gonzalez Garcia ME , et al. Comparative effectiveness of teclistamab versus real‐world physician’s choice of therapy in LocoMMotion and MoMMent in triple‐class exposed relapsed/refractory multiple myeloma. Adv Ther. 2024;41(2):696‐715. doi:10.1007/s12325-023-02738-0 PMC10838813 38110653

[74] Costa L , LeBlanc TW , Tesch H , et al. An updated indirect comparison of elranatamab versus a real‐world external control arm in triple‐class refractory multiple myeloma. Blood Lymphat Cancer. 2025;15:11‐20. doi:10.2147/blctt.s516356 PMC12147782 40491859

[75] Tsang C , O’Reilly JE , Carpenter L , et al. Comparison of outcomes with elranatamab and real world treatments in the UK for triple class exposed relapsed and refractory multiple myeloma. BMC Cancer. 2025;25(1):1219. doi:10.1186/s12885-025-14624-9 PMC12297639 40713551

[76] Mol I , Hu Y , LeBlanc TW , et al. A matching‐adjusted indirect comparison of the efficacy of elranatamab versus physician’s choice of treatment in patients with triple‐class exposed/refractory multiple myeloma. Curr Med Res Opin. 2024;40(2):199‐207. doi:10.1080/03007995.2023.2277850 38078866

[77] Mol I , Hu Y , LeBlanc TW , et al. Elranatamab versus physician’s choice of treatment in patients with triple‐class exposed/refractory multiple myeloma: an updated matching‐adjusted indirect comparison. J Comp Eff Res. 2025;14(5):e240236. doi:10.57264/cer-2024-0236 PMC12007480 40190254

[78] Kumar S , Jagannath S , Weisel KC , et al. Linvoseltamab versus real‐world International Myeloma Working Group standard‐of‐care in triple‐class exposed relapsed/refractory multiple myeloma. Blood Cancer J. 2026;16(1):44. doi:10.1038/s41408-026-01466-2 PMC13031307 41881954

[79] Raje NS , Leleu XP , Lesokhin AM , et al. Efficacy and safety of elranatamab by age and frailty in patients (pts) with relapsed/refractory multiple (RRMM): a subgroup analysis from MagnetisMM‐3 [abstract]. J Clin Oncol. 2023;41(16 suppl):8040. doi:10.1200/jco.2023.41.16_suppl.8040

[80] Adegbite B , Tan CR , Shekarkhand T , et al. Outcomes in frail patients receiving BCMA‐directed bispecific antibodies for relapsed/refractory multiple myeloma. Blood Adv. 2025;9(15):4016‐4022. doi:10.1182/bloodadvances.2025015973 PMC12345270 40315372

[81] Coste A , Godet S , Manier S , et al. Teclistamab in elderly patients with relapsed/refractory multiple myeloma: a subgroup analysis of the French RetrosTECtive cohort. EJHaem. 2026;7(1):e70233. doi:10.1002/jha2.70233 PMC12854094 41623549

[82] Dima D , Vazquez‐Martinez MA , Davis JA , et al. Outcomes of teclistamab (Tec) in patients with relapsed/refractory multiple myeloma (RRMM) with prior exposure to BCMA‐directed therapy (BCMA‐DT): a multicenter study from the U.S. Multiple Myeloma Immunotherapy Consortium [abstract]. Blood. 2024;144(suppl 1):897. doi:10.1182/blood-2024-203409 PMC12198414 40562770

[83] Costa LJ , Banerjee R , Mian H , et al. International Myeloma Working Group Immunotherapy Committee recommendation on sequencing immunotherapy for treatment of multiple myeloma. Leukemia. 2025;39(3):543‐554. doi:10.1038/s41375-024-02482-6 PMC11879857 39870767

[84] van de Donk NWCJ , Moreau P , San‐Miguel JF , et al. Sequencing BCMA‐ and GPRC5D‐targeting immunotherapies in multiple myeloma: practical guidance from the European Myeloma Network. Hemasphere. 2025;9(11):e70260. doi:10.1002/hem3.70260 PMC12645799 41306326

[85] Li J , Piskol R , Ybarra R , et al. CD3 bispecific antibody‐induced cytokine release is dispensable for cytotoxic T cell activity. Sci Transl Med. 2019;11(508):eaax8861. doi:10.1126/scitranslmed.aax8861 31484792

[86] Rodriguez‐Otero P , Usmani S , Cohen AD , et al. International Myeloma Working Group Immunotherapy Committee consensus guidelines and recommendations for optimal use of T‐cell–engaging bispecific antibodies in multiple myeloma. Lancet Oncol. 2024;25(5):e205‐e216. doi:10.1016/s1470-2045(24)00043-3 38697166

[87] Lund T , Khandanpour C , Anguille S , et al. European expert panel consensus on outpatient administration of teclistamab and talquetamab in patients with multiple myeloma: feasibility, key considerations, and future directions. Eur J Haematol. 2025;115(4):358‐366. doi:10.1111/ejh.14444 PMC12402850 40571970

[88] Garfall AL , Banerjee R , Frenzel L , et al. A roadmap to implementing outpatient administration of bispecific antibodies in multiple myeloma. Front Oncol. 2025;15:1630146. doi:10.3389/fonc.2025.1630146 PMC12343214 40809030

[89] Rodríguez‐Otero P , Moreau P , Mateos MV , et al. European expert recommendations on teclistamab management for relapsed or refractory multiple myeloma. Clin Lymphoma Myeloma Leuk. 2026;26(3):e408‐e420. doi:10.1016/j.clml.2025.12.003 41577552

[90] Van De Donk NWCJ , Garfall AL , Benboubker L , et al. Longer‐term follow‐up of patients (pts) receiving prophylactic tocilizumab (toci) for the reduction of cytokine release syndrome (CRS) in the phase 1/2 MajesTEC‐1 study of teclistamab in relapsed/refractory multiple myeloma (RRMM) [abstract]. J Clin Oncol. 2024;42(16 suppl):7517. doi:10.1200/jco.2024.42.16_suppl.7517

[91] Rifkin R , Schade H , Simmons G , et al. Optec: a phase 2 study to evaluate outpatient step‐up administration of teclistamab in patients with relapsed/refractory multiple myeloma (RRMM): updated results [abstract]. Blood. 2024;144(suppl 1):4753. doi:10.1182/blood-2024-207295

[92] Schinke C , Vij R , Jagannath S , et al. MM‐216: Prophylactic tocilizumab to mitigate cytokine release syndrome and outpatient dosing of talquetamab in relapsed/refractory multiple myeloma: updated phase 1/2 Monumental‐1 results [abstract]. Clin Lymphoma Myeloma Leuk. 2025;25(suppl 1):S908. doi:10.1016/s2152-2650(25)02580-7

[93] Kowalski A , Lykon J , Diamond B , et al. Tocilizumab prophylaxis for patients with multiple myeloma treated with bispecific antibodies. Blood Adv. 2025;9(19):4979‐4986. doi:10.1182/bloodadvances.2025016911 PMC12514479 40590849

[94] Lee DW , Santomasso BD , Locke FL , et al. ASTCT consensus grading for cytokine release syndrome and neurologic toxicity associated with immune effector cells. Biol Blood Marrow Transplant. 2019;25(4):625‐638. doi:10.1016/j.bbmt.2018.12.758 PMC12180426 30592986

[95] Martin TG , Mateos MV , Nooka A , et al. Detailed overview of incidence and management of cytokine release syndrome observed with teclistamab in the MajesTEC‐1 study of patients with relapsed/refractory multiple myeloma. Cancer. 2023;129(13):2035‐2046. doi:10.1002/cncr.34756 36991547

[96] Mancuso K , Talarico M , Manzato E , et al. T‐cell redirecting therapies in multiple myeloma: pathogenesis and management of toxicities beyond CRS and ICANS. Cancers (Basel). 2025;17(9):1514. doi:10.3390/cancers17091514 PMC12070925 40361441

[97] Reynolds G , Cliff ERS , Mohyuddin GR , et al. Infections following bispecific antibodies in myeloma: a systematic review and meta‐analysis. Blood Adv. 2023;7(19):5898‐5903. doi:10.1182/bloodadvances.2023010539 PMC10558589 37467036

[98] Jourdes A , Cellerin E , Touzeau C , et al. Characteristics and incidence of infections in patients with multiple myeloma treated by bispecific antibodies: a national retrospective study. Clin Microbiol Infect. 2024;30(6):764‐771. doi:10.1016/j.cmi.2024.02.023 38432433

[99] Lancman G , Parsa K , Kotlarz K , et al. IVIg use associated with ten‐fold reduction of serious infections in multiple myeloma patients treated with anti‐BCMA bispecific antibodies. Blood Cancer Discov. 2023;4(6):440‐451. doi:10.1158/2643-3230.bcd-23-0049 PMC10618720 37769148

[100] Hammons LR , Szabo A , Janardan A , et al. Kinetics of humoral immunodeficiency with bispecific antibody therapy in relapsed refractory multiple myeloma. JAMA Netw Open. 2022;5(10):e2238961. doi:10.1001/jamanetworkopen.2022.38961 PMC9617177 36306135

[101] Cani L , Scott SA , Roberts D , et al. Infection risk in 158 patients with relapsed/refractory multiple myeloma treated with bispecific antibodies: a single‐center experience. Haematologica. 2025;111:656‐664. doi:10.3324/haematol.2025.288187 PMC12862324 40905083

[102] Mazahreh F , Mazahreh L , Schinke C , et al. Risk of infections associated with the use of bispecific antibodies in multiple myeloma: a pooled analysis. Blood Adv. 2023;7(13):3069‐3074. doi:10.1182/bloodadvances.2022009435 PMC10331406 36857755

[103] Jelinek T , Zihala D , Zabaleta A , et al. Selective B‐cell subset depletion underlies increased infection risk in patients with MM treated with anti‐BCMA vs anti‐GPRC5D bsAbs. Blood. 2026;147(10):1070‐1082. doi:10.1182/blood.2025029572 PMC13014095 41405507

[104] Frerichs KA , Verkleij CPM , Mateis MV , et al. Teclistamab impairs humoral immunity in patients with heavily pretreated myeloma: importance of immunoglobulin supplementation. Blood Adv. 2024;8(1):194‐206. doi:10.1182/bloodadvances.2023011658 PMC10787247 38052042

[105] Mohan M , Szabo A , Cheruvalath H , et al. Effect of intravenous immunoglobulin (IVIG) supplementation on infection‐free survival in recipients of BCMA‐directed bispecific antibody therapy for multiple myeloma. Blood Cancer J. 2025;15(1):74. doi:10.1038/s41408-025-01282-0 PMC12018942 40268898

[106] Piron B , Rodrigo L , Tessoulin B , et al. Preemptive immunoglobulin prophylaxis reduces infections in patients treated with anti‐BCMA bispecific antibodies. Clin Lymphoma Myeloma Leuk. 2026;26(2):115‐122.e6. doi:10.1016/j.clml.2025.10.024 41274822

[107] Smits F , Groen K , Korst CLBM , et al. Immunoglobulin supplementation and longer dosing intervals reduce risk of infections in patients with RRMM treated with teclistamab. Blood Cancer J. 2026;16(1):26. doi:10.1038/s41408-026-01451-9 PMC12960722 41720763

[108] Ludwig H , Munshi NC , Terpos E , et al. Proposal for harmonizing the reporting of infections during treatment with bispecific antibodies in multiple myeloma. Blood Adv. 2024;8(18):4979‐4982. doi:10.1182/bloodadvances.2024013461 PMC11496977 38776433

[109] Nooka AK , Rodriguez C , Mv M , et al. Incidence, timing, and management of infections in patients receiving teclistamab for the treatment of relapsed/refractory multiple myeloma in the MajesTEC‐1 study. Cancer. 2023;130(6):886‐900. doi:10.1002/cncr.35107 37960969

[110] Ocio EM , Mateos MV , Magen H , et al. P38 | Safety and efficacy of talquetamab + teclistamab in patients with relapsed/refractory multiple myeloma from phase 1b of RedirecTT‐1: results with an extended median follow‐up of 3 years. Haematologica. 2026;111(S2):P38. doi:10.3324/haematol.2026.s2.14054

[111] Costa LJ , Bahlis NJ , Perrot A , et al. Teclistamab plus daratumumab in relapsed or refractory multiple myeloma. N Engl J Med. 2026;394(8):739‐752. doi:10.1056/nejmoa2514663 PMC13218738 41363801

[112] Touzeau C , Mina R , Quach H , et al. Teclistamab in multiple myeloma with one to three previous lines of therapy. N Engl J Med. 2026;395(5):440‐453. doi:10.1056/NEJMoa2603870 42212933

[113] Raje N , Anderson K , Einsele H , et al. Monitoring, prophylaxis, and treatment of infections in patients with MM receiving bispecific antibody therapy: consensus recommendations from an expert panel. Blood Cancer J. 2023;13(1):116. doi:10.1038/s41408-023-00879-7 PMC10394080 37528088

[114] Mohan M , Chakraborty R , Bal S . Recommendations on prevention of infections during chimeric antigen receptor T‐cell and bispecific antibody therapy in multiple myeloma. Br J Haematol. 2023;203(5):736‐746. 10.1111/bjh.18909 PMC10700672 37287117

[115] Cheema H , Shrestha A , Naqvi S , et al. Clinical outcomes and risk factors of cytomegalovirus reactivation in teclistamab‐treated multiple myeloma patients. Blood Cancer J. 2026;16(1):51. doi:10.1038/s41408-026-01484-0 PMC13039453 41916955

[116] Banerjee R , Mohan M , Rejeski K , et al. Immunoglobulin prophylaxis should be initiated after bispecific antibody therapy in multiple myeloma, regardless of IgG levels. Blood Adv. 2025;9(18):4720‐4726. doi:10.1182/bloodadvances.2025016490 PMC12466225 40690761

[117] Frerichs KA , Broekmans ME , Marin Soto JA , et al. Preclinical activity of JNJ‐7957, a novel BCMA×CD3 bispecific antibody for the treatment of multiple myeloma, is potentiated by daratumumab. Clin Cancer Res. 2020;26(9):2203‐2215. doi:10.1158/1078-0432.ccr-19-2299 31969333

[118] Cho SF , Lin L , Xing L , et al. The immunomodulatory drugs lenalidomide and pomalidomide enhance the potency of AMG 701 in multiple myeloma preclinical models. Blood Adv. 2020;4(17):4195‐4207. doi:10.1182/bloodadvances.2020002524 PMC7479960 32898244

[119] Paiva B , Gaffney B , Burnett K , et al. P799: Synergistic antitumor activity of the BCMA 2 + 1 T cell engager (TCE) alnuctamab (ALNUC; BMS‐986349; CC‐93269) and celmod agents in multiple myeloma (MM) preclinical models [abstract]. Hemasphere. 2023;7(suppl):e805462a. doi:10.1097/01.hs9.0000970100.80546.2a

[120] Zamagni E , Silzle T , Špička I , et al. Phase 3 study of teclistamab (Tec) in combination with lenalidomide (Len) and Tec alone versus Len alone in newly diagnosed multiple myeloma (NDMM) as maintenance therapy following autologous stem cell transplantation (ASCT): safety run‐in (SRI) results from the Majestec‐4/EMN30 trial [abstract]. Blood. 2024;144(suppl):494. doi:10.1182/blood-2024-205608

[121] Raab MS , Weinhold N , Kortüm KM , et al. Teclistamab‐based induction treatment in transplant‐eligible, newly diagnosed multiple myeloma: a phase 2 trial. Nat Med. 2026;32(7):2440‐2448. doi:10.1038/s41591-026-04471-x PMC13375549 42350642

[122] Kazandjian D , Diamond B , Hoffman J , et al. A phase 2 trial of abbreviated fixed‐duration (default 4 cycles) linvoseltamab immuno‐consolidation to deepen responses post newly diagnosed multiple myeloma combination therapy for minimal residual disease positivity (the IMMUNOPLANT study (abstract). Blood. 2025;146(suppl 1):248. doi:10.1182/blood-2025-248

[123] Touzeau C , Beksac M , Terpos E , et al. Safety results from the phase 3 MajesTEC‐7 study in patients (pts) with transplant ineligible/not intended newly diagnosed multiple myeloma (NDMM). J Clin Oncol. 2024;42(16 suppl):7506. doi:10.1200/jco.2024.42.16_suppl.7506

[124] Manier S , Lambert J , Macro M , et al. A phase 2 study of teclistamab in combination with daratumumab in elderly patients with newly diagnosed multiple myeloma: the IFM2021‐01 Teclille trial, cohort A [abstract]. Blood. 2025;146(suppl 1):367. doi:10.1182/blood-2025-367

[125] Searle E , Quach H , Wong S , et al. P30 Single cohort results from MajesTEC‐2: teclistamab (TEC) in combination with subcutaneous daratumumab (DARA) and lenalidomide (LEN) in patients with multiple myeloma (MM). Hemasphere. 2023;7(S2):27. doi:10.1097/01.hs9.0000936248.01150.e8

[126] Tan C , Searle E , Anguille S , et al. P865: Teclistamab in combination with lenalidomide in previously treated patients with multiple myeloma in the phase 1b multicohort MajesTEC‐2 study [abstract]. Hemasphere. 2023;7(suppl 3):e162590e. doi:10.1097/01.hs9.0000970364.16259.0e

[127] D’Souza A , Costa LJ , San‐Miguel JF , et al. Teclistamab, daratumumab, and pomalidomide in patients with relapsed/refractory multiple myeloma: results from the MajesTEC‐2 cohort A and Trimm‐2 studies [abstract]. Blood. 2024;144(suppl 1):495‐497. doi:10.1182/blood-2024-200181

[128] Rodriguez Otero P , D’Souza A , Reece D , et al. S188: Teclistamab in combination with daratumumab, a novel, immunotherapy‐based approach for the treatment of relapsed/refractory multiple myeloma: updated phase 1b results [abstract]. Hemasphere. 2022;6:89‐90. doi:10.1097/01.hs9.0000843644.02496.66

[129] Suvannasankha A , Kaufman J , Badros A , et al. Safety and efficacy of elranatamab in combination with iberdomide in patients with relapsed or refractory multiple myeloma: results from the phase 1b MagnetisMM‐30 trial [abstract]. Blood. 2025;146(suppl 1):100. doi:10.1182/blood-2025-100

[130] Byun JM , Min CK , Kim K , et al. Phase I/II study of mezigdomide and elranatamab for relapsed/refractory multiple myeloma patients (MELT‐MM): initial results from part 1 [abstract]. Blood. 2025;146(suppl 1):5835. doi:10.1182/blood-2025-5835

[131] Cohen YC , Magen H , Gatt M , et al. Talquetamab plus teclistamab in relapsed or refractory multiple myeloma. N Engl J Med. 2025;392(2):138‐149. doi:10.1056/nejmoa2406536 39778168

[132] Kumar S , Mateos MV , Ye JC , et al. Dual targeting of extramedullary myeloma with talquetamab and teclistamab. N Engl J Med. 2026;394(1):51‐61. doi:10.1056/nejmoa2514752 41358582

[133] Banerjee R , van de Donk NWCJ , Mina R , et al. Teclistamab plus daratumumab (Tec‐Dara) in patients (Pts) with relapsed refractory multiple myeloma (RRMM). Analysis of MajesTEC‐3 based on cytogenetic and functional risk. Hemasphere. 2026;10(S1):232‐234. doi:10.1002/hem3.70419

[134] Harrison SJ , Minnema MC , Lee HC , et al. A phase 1 first in human (FIH) study of AMG 701, an anti‐B‐cell maturation antigen (BCMA) half‐life extended (HLE) BiTE® (bispecific T‐cell engager) molecule, in relapsed/refractory (RR) multiple myeloma (MM) [abstract]. Blood. 2020;136(suppl 1):28‐29. doi:10.1182/blood-2020-134063

[135] Bar N , Martin T , Hofmeister CC , et al. Alnuctamab, a bivalent B‐cell maturation antigen–targeting T cell engager for patients with relapsed or refractory multiple myeloma: results from a phase 1, first‐in‐human study. Leukemia. 2026;40(3):481‐490. doi:10.1038/s41375-025-02841-x PMC12960249 41501501

[136] Suvannasankha A , Kapoor P , Pianko MJ , et al. Abstract CT013: Safety and efficacy from the phase 1/2 first‐in‐human study of REGN5459, a BCMA×CD3 bispecific antibody with low CD3 affinity, in patients with relapsed/refractory multiple myeloma. Cancer Res. 2023;83(8 suppl):CT013. doi:10.1158/1538-7445.am2023-ct013

[137] An G , Huang Z , Liang A , Fang B , Jing H , Qiu LG . Anti‐CD3/B‐cell maturation antigen bispecific antibody CM336 in relapsed/refractory multiple myeloma: an open‐label, phase I/II study. Blood. 2024;144(suppl 1):3377. doi:10.1182/blood-2024-206742

[138] Baljevic M , Voorhees PM , Valdes CR , et al. Long‐term efficacy and safety of etentamig, a B‐cell maturation antigen (BCMA) bispecific antibody in patients with relapsed/refractory multiple myeloma (RRMM) [abstract]. J Clin Oncol. 2025;43(16 suppl):7527. doi:10.1200/JCO.2025.43.16_suppl.7527

[139] Rodriguez Valdes C , Voorhees PM , D'Souza A , et al. Efficacy, safety, and determination of RP2D of ABBV‐383, a BCMA bispecific antibody, in patients with relapsed/refractory multiple myeloma (RRMM) [abstract]. J Clin Oncol. 2024;42(16 suppl):7531. doi:10.1200/jco.2024.42.16_suppl.7531

[140] Rodriguez C , Mielnik M , Kazandjian D , et al. ABBV‐383 plus daratumumab‐dexamethasone in relapsed or refractory multiple myeloma: a phase 1b dose‐escalation and safety expansion study [abstract]. Blood. 2024;144(suppl 1):496. doi:10.1182/blood-2024-205083

[141] Voorhees P , D'Souza A , Quach H , et al. Etentamig plus pomalidomide‐dexamethasone combination therapy in relapsed or refractory multiple myeloma: a phase 1b dose‐escalation and safety expansion study [abstract]. Blood. 2025;146(suppl 1):247. doi:10.1182/blood-2025-247

[142] Hipp S , Tai YT , Blanset D , et al. A novel BCMA/CD3 bispecific T‐cell engager for the treatment of multiple myeloma induces selective lysis in vitro and in vivo. Leukemia. 2017;31(8):1743‐1751. doi:10.1038/leu.2016.388 28025583

[143] Topp MS , Duell J , Zugmaier G , et al. Anti–B‐cell maturation antigen BiTE molecule AMG 420 induces responses in multiple myeloma. J Clin Oncol. 2020;38(8):775‐783. doi:10.1200/jco.19.02657 31895611

[144] Tacchetti P , Barbato S , Mancuso K , Zamagni E , Cavo M . Bispecific antibodies for the management of relapsed/refractory multiple myeloma. Cancers (Basel). 2024;16(13):2337. doi:10.3390/cancers16132337 PMC11240369 39001399

[145] Goldstein RL , Goyos A , Li CM , et al. AMG 701 induces cytotoxicity of multiple myeloma cells and depletes plasma cells in cynomolgus monkeys. Blood Adv. 2020;4(17):4180‐4194. doi:10.1182/bloodadvances.2020002565 PMC7479952 32886754

[146] Seckinger A , Delgado JA , Moser S , et al. Target expression, generation, preclinical activity, and pharmacokinetics of the BCMA‐T cell bispecific antibody EM801 for multiple myeloma treatment. Cancer Cell. 2017;31(3):396‐410. doi:10.1016/j.ccell.2017.02.002 28262554

[147] Buelow B , Choudry P , Clarke S , et al. Pre‐clinical development of TNB‐383B, a fully human T‐cell engaging bispecific antibody targeting BCMA for the treatment of multiple myeloma [abstract]. J Clin Oncol. 2018;36(15 suppl):8034. doi:10.1200/jco.2018.36.15_suppl.8034

[148] Valdes CR , Lichtman E , Voorhees P , et al. Infection rate profile of etentamig monotherapy in patients with relapsed/refractory multiple myeloma. Poster presented at: 12th World Congress on Controversies in Multiple Myeloma (COMy); May 14–17, 2025; Paris, France. Accessed June 15, 2026. https://comy.cme‐congresses.com/wp‐content/uploads/2026/05/54‐eposter‐2026.pdf

[149] Foureau DM , Bhutani M , Robinson M , et al. Ex vivo efficacy of BCMA‐bispecific antibody TNB‐383B in relapsed/refractory multiple myeloma. EJHaem. 2020;1:113‐121. doi:10.1002/jha2.69 PMC9175895 35847733

[150] Ran W , Sun X , Yang J . BTL‐101, a fully human trispecific BCMA×GPRC5D×CD3 T‐cell engager for the treatment of multiple myeloma [abstract]. Hemasphere. 2026;10(S1):S189.

[151] Nadeem O Teclistamab improves depth of response and PFS versus lenalidomide‐dexamethasone in high‐risk smoldering multiple myeloma: results from the phase 2 IMMUNOPRISM trial. Nat Med. Published online September 11, 2026. doi:10.1038/s41591-026-04642-w PMC13668702 42728379

[152] Broijl A , Schjesvold F , Cerchione C , et al. Safety and efficacy of elranatamab as early intervention in patients with high‐risk smoldering myeloma: first results from the phase 2 ERASMM (EMN34) study [abstract]. Hemasphere. 2026;10(S1):S205.

[153] Rodríguez‐Otero P , Bargay Lleonart JJ , Clavero ME , et al. Safety and efficacy of linvoseltamab (LINVO) in patients (Pts) with high‐risk smoldering multiple myeloma (HR‐SMM): first results from the phase 2 LINKER‐SMM1 trial [abstract]. Clin Lymphoma Myeloma Leuk. 2025;25:S366‐S367. doi:10.1016/s2152-2650(25)04011-x

[154] Bal S , Sigler A , Chan A , et al. First description of B cell maturation antigen expression in light chain amyloidosis [abstract]. Blood. 2019;134(suppl 1):5452. doi:10.1182/blood-2019-127332

[155] Godara A , Zhou P , Kugelmass A , et al. Presence of soluble and cell‐surface B‐cell maturation antigen in systemic light‐chain amyloidosis and its modulation by gamma‐secretase inhibition. Am J Hematol. 2020;95(5):E110‐E113. doi:10.1002/ajh.25734 31951032

[156] Mann H , Toskic D , Pak K , Willard P , Fogaren T , Comenzo R . Teclistamab induces rapid responses in patients with relapsed/refractory AL amyloidosis [abstract]. Blood. 2023;142(suppl 1):6766. doi:10.1182/blood-2023-182971

[157] Leung N , Chapman JA , Bhatia S . First report of teclistamab in a patient with relapsed AL amyloidosis and multiple myeloma. eJHaem. 2023;4:1157‐1159. doi:10.1002/jha2.772 PMC10660095 38024617

[158] Chakraborty R , Bhutani D , Maurer MS , Mohan M , Lentzsch S , D’Souza A . Safety and efficacy of teclistamab in systemic immunoglobulin light chain amyloidosis. Blood Cancer J. 2023;13(1):172. doi:10.1038/s41408-023-00950-3 PMC10682473 38012151

[159] Stalker M , Garfall A , Cohen A , et al. Safety and efficacy of teclistamab in patients with relapsed or refractory AL amyloidosis. Eur J Haematol. 2025;114(3):443‐447. doi:10.1111/ejh.14348 PMC11798760 39552142

[160] Forgeard N , Elessa D , Carpinteiro A , et al. Teclistamab in relapsed or refractory AL amyloidosis: a multinational retrospective case series. Blood. 2024;143(8):734‐737. doi:10.1182/blood.2023022937 38096365

[161] Vianna P , Chakraborty R , Hossain S , et al. Safety and efficacy of elranatamab in patients with relapsed and/or refractory immunoglobulin light‐chain amyloidosis. Blood. 2025;146(16):1929‐1935. doi:10.1182/blood.2025028383 PMC12757077 40712156

[162] Rees MJ , Khouri J , Grajales‐Cruz AF , et al. The real‐world safety and efficacy of bispecific T‐cell engager therapy in systemic AL amyloidosis. Am J Hematol. 2026;101(1):187‐192. doi:10.1002/ajh.70140 41221958

[163] Chakraborty R , Kastritis E , Huart A , et al. Phase 1/2 dose escalation and expansion study of etentamig in patients with relapsed or refractory light chain amyloidosis [abstract]. Blood. 2025;146(suppl 1):692. doi:10.1182/blood-2025-692

[164] Talbot A , Forgeard N , Vaugeois T , et al. Teclistamab for heavily pretreated relapsed/refractory POEMS syndrome. Haematologica. 2025;111(4):1490‐1493. doi:10.3324/haematol.2025.287606 PMC13040146 40605704

[165] Battaglini J , Henderson J , Sanchorawala V , Szalat R . Teclistamab therapy for refractory type 1 cryoglobulinemia. Haematologica. 2025;110(8):1891‐1893. doi:10.3324/haematol.2025.287313 PMC12358774 40109179

[166] Düsing C , Györfi AH , Stütz AN , et al. Bispecific T cell engagers for treatment‐refractory autoimmune connective tissue diseases. Nat Med. 2026;32(4):1530‐1542. doi:10.1038/s41591-026-04238-4 PMC13099420 41714747

